# Enriching stabilizing mutations through automated analysis of molecular dynamics simulations using BoostMut

**DOI:** 10.1002/pro.70334

**Published:** 2025-10-11

**Authors:** Kerlen T. Korbeld, Maximilian J. L. J. Fürst

**Affiliations:** ^1^ Molecular Enzymology University of Groningen Groningen The Netherlands

**Keywords:** energy calculations, machine learning, MD simulations, protein engineering, thermostability

## Abstract

Thermostability is a critical goal in protein engineering for applications of biocatalysts and biomedicines. Despite striking advances in biomolecular predictive modeling, reliably identifying stabilizing mutations remains challenging. Previously, molecular dynamics (MD) simulations and visual inspection have been used as a secondary filter to improve the success rate of mutations pre‐selected by thermostability algorithms. However, this approach suffers from low throughput and subjectivity. Here, we introduce Biophysical Overview of Optimal Stabilizing Mutations (BoostMut), a computational tool that standardizes and automates mutation filtering by analyzing dynamic structural features from MD. BoostMut formalizes the principles guiding manual verification, providing a consistent and reproducible stability assessment. Rigorous benchmarking across multiple datasets showed that integrating BoostMut's biophysical analysis improves the prediction rate regardless of the initial thermostability predictor. Given a modest amount of existing mutant stability data, BoostMut's performance can be further enhanced with a lightweight machine learning model. Upon experimentally validating BoostMut predictions on the enzyme limonene epoxide hydrolase, we identified stabilizing mutations previously overlooked by visual inspection and achieved a higher overall success rate. We foresee BoostMut being used for mutation filtering, as an integrated step in thermostability prediction workflows, and for labeling data to train future predictors.

## 1 INTRODUCTION

Proteins are widely utilized in biotechnological and medical applications outside their natural environment, often causing suboptimal performance (da Silva et al., 2022). Enzymes, for instance, employed in biocatalysis and synthetic biology, frequently exhibit reduced activity and specificity when acting on non‐natural substrates. Another critical limitation in protein applications is instability, causing, for example, low space–time yields in bioconversions or rapid clearance of biological drugs (Woodley, 2013; Zaman et al., 2019). Moreover, unstable proteins are poor starting points for enzyme engineering campaigns aimed at enhancing catalytic properties, as such efforts often require the introduction of functionally beneficial, yet destabilizing mutations (Bloom et al., 2006).

A solution to mitigate challenges with instability lies in the use of thermostable proteins, which additionally offer the advantage of being easily enriched or purified through heat denaturation of the expression host's background proteome (Olichon et al., 2007; Schenkel et al., 2021). While naturally stable homologs from thermophilic organisms are sometimes available, many mesostable homologs display unique functions (Nguyen et al., 2024). Furthermore, extensive prior characterizations of non‐stable proteins may discourage switching to new homologs or even de novo designed variants. Therefore, engineering existing proteins for enhanced stability is often preferred.

The stabilizing effect of a candidate mutation is generally quantified through either the melting temperature (*T*_m_) obtained from a thermal shift assay, or the Gibbs free energy of unfolding (Δ*G*), with the final values reported as a difference between the wild type and mutant (Δ*T*_m_ and ΔΔ*G*, respectively) (Senisterra & Finerty, 2009). Among the methods developed to identify such changes, the simplest approach—random mutagenesis followed by screening—primarily yields destabilizing mutations. Although a universal base rate is difficult to determine, estimates based on aggregated stability data suggest that stabilizing mutations (defined as Δ*T*_m_ >0°C or ΔΔ*G*< 0 kcal/mol) occur at a frequency of 20%–30% (Louis & Abriata, 2021; Taverna & Goldstein, 2002; Tokuriki et al., 2007), although this frequency drops to only 3%–5% when neutral mutations (defined as ΔΔ*G* > −1 or <1 kcal/mol) are binned separately (Nisthal et al., 2019; Tokuriki & Tawfik, 2009). Notably, the practical success rate is lowered even further: for instance, stabilizing the often energetically unfavorable geometries in catalytic sites (Modarres et al., 2016; Shoichet et al., 1995) compromises activity and is thus undesirable. Moreover, because approximately 30% of random mutations impair protein expression by impacting folding or solubility and thus prevent measurement (Velecký et al., 2022), stability datasets likely inflate the apparent proportion of stabilizing mutations.

To improve the identification of stabilizing mutations, numerous computational methods have been developed. Most rely on training or calibrating algorithms on datasets of empirically measured thermostability changes, compiled in various thermostability databases (Bava et al., 2004; Stourac et al., 2020; Xavier et al., 2020). While most energy calculations‐based predictors are tuned with these datasets (Guerois et al., 2002; Kellogg et al., 2011; Parthiban et al., 2006), other methods incorporate evolutionary data (Montanucci et al., 2022; Pires et al., 2014). Recent advances in machine learning (ML) have further enabled the integration of structural and evolutionary information from large databases like UniProt and the PDB, rekindling the quest for the long‐sought “perfect” stability predictor (Diaz et al., 2024; Jiang et al., 2024; Sun et al., 2023).

Yet, despite the availability of large datasets and continuous algorithmic improvements, the overrepresentation of destabilizing mutations in the data (Caldararu et al., 2020) is the likely cause of a persistent significantly lower accuracy with stabilizing mutations, compared to destabilizing ones (Benevenuta et al., 2023; Diaz et al., 2024). A compounding issue in the field is the use of correlation‐based metrics and overall accuracy, which fail to account for this imbalance (Broom et al., 2020). The FoldX tool exemplifies this problem: despite strong benchmark performance and widespread use (Buß et al., 2018; Gerasimavicius et al., 2023; Velecký et al., 2024), its predicted stabilizing mutations have an experimental success rate of only ~29%, while destabilizing mutations are identified correctly ~69% of the time (Buß et al., 2018). Similarly, the specificity (accuracy for destabilizing mutations) of most other predictors is about twice as high as sensitivity (accuracy for stabilizing mutations) (Khan & Vihinen, 2010). Even a state‐of‐the‐art method's large language model predictor only achieved a 44% success rate (45/103) in predicting stabilizing mutation (Jiang et al., 2024).

In light of these issues, many protein engineering campaigns aim to improve the success rate by combining the primary predictor with a filter step, leveraging the strength of the former in eliminating destabilizing mutations, while applying additional criteria for refinement. In its most simple and common form, a manual, typically visual, inspection assesses mutations before experimental testing to catch obvious errors. This often overlooked, because understated part of computational engineering pipelines heavily relies on the researcher's biophysical expertise (Borgo & Havranek, 2012; Fischer et al., 2021; Lippow & Tidor, 2007). A more advanced filtering mechanism has been proposed in the Framework for Rapid Enzyme Stabilization by Computational Libraries (FRESCO), which uses FoldX and Rosetta to generate a favorable mutation set, followed by high‐throughput molecular dynamics (MD) simulations and visual inspection to incorporate structural dynamics (Wijma et al., 2014, 2018). This method has consistently led to substantial thermostability gains, with one campaign producing a 10‐fold mutant exhibiting a Δ*T*_m_ of +51°C (Aalbers et al., 2020).

However, although visual inspection improves thermostability predictors' success rates, it remains limited by subjectivity, labor intensity, and low throughput. The reliance on human judgment constrains speed, scale, and reproducibility, making systematic high‐throughput screening impractical. To overcome this issue, we developed Biophysical Overview of Optimal Stabilizing Mutations (BoostMut), a secondary filter that analyzes structural features of MD‐simulated mutations. BoostMut formalizes the inspection principles, providing a consistent and reproducible method for evaluating mutations generated by primary predictors. BoostMut outputs a set of interpretable biophysical metrics that can rationalize the effect of mutations and serve as structured input to train ML models. We evaluated BoostMut on 1584 mutations across six proteins and established that it outperforms visual inspection and commonly used predictor scores in ranking. We demonstrate its effectiveness by experimentally analyzing 18 previously untested mutations in limonene epoxide hydrolase and show that BoostMut filtering selects stabilizing mutations with a success rate of 46% in this protein.

## 2 RESULTS

**FIGURE 1:**
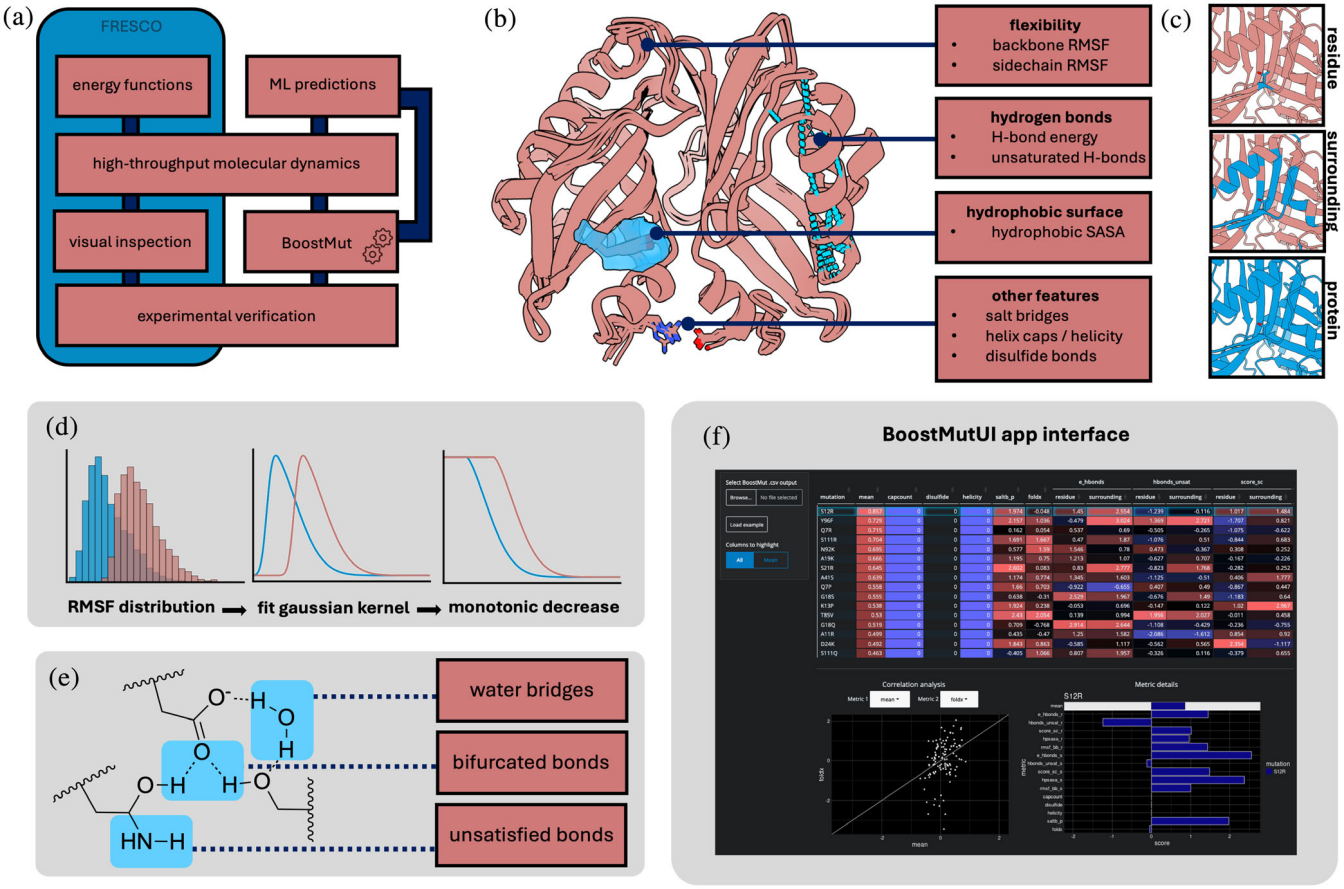
(a) Overview of the FRESCO and BoostMut pipelines. (b) The BoostMut metrics. (c) Metrics are evaluated at the residue, 8 Å surrounding, or the entire protein level. (d) Evaluation of protein hydrogen bonding distinguishes water‐mediated H bonds, bifurcated H bonds, and unsaturated donor/acceptors. (e) To obtain benchmark curves, the distribution of the property is fitted using a Gaussian kernel density estimation, and a monotonic decrease is guaranteed. (f) BoostMut's online user interface visualizing the output.

At its core, BoostMut analyses various biophysical properties of protein structure ensembles such as MD trajectories, to compare the stability of a set of mutants to the wild type. While running even short MDs for all possible single mutants would typically be too computationally expensive, it becomes feasible once a narrower set of mutations has already been pre‐selected. MDs and BoostMut are therefore specifically meant as a filter after already having selected mutations by a given primary predictor (Figure 1a).

The metrics analyzed by BoostMut are based on physical properties of proteins known to affect thermostability and widely used as screening criteria when assessing mutations (Floor et al., 2014; Wijma et al., 2018). They mainly comprise improvement of the hydrogen bond (H bond) network via increased intramolecular or reduced unsatisfied donor/acceptor groups, prevention of increased protein flexibility, and minimization of solvent‐exposed hydrophobic residues (Figure 1b). BoostMut encapsulates these and other criteria into a formalized set of metrics, whose output can be used for scoring and ranking, or as input for ML methods.

We implemented our analyses via the MDAnalysis python library (Gowers et al., 2016), which provides a framework for analyzing MD trajectories and allows BoostMut to work across different topology and trajectory formats. For each metric, the difference between the mutant and the wild type, averaged over each trajectory frame, is reported. BoostMut always acts at three distinct levels of granularity: the mutated residue itself, its local environment, or taking the entire protein into account. For each selection, a more complete effect of the mutation on its surroundings is obtained, but also the amount of noise increases (Figure 1c).

The first metric we assess is hydrogen bonding, which is known to increase protein stability by decreasing the enthalpy of the folded state (Pace et al., 2014). Yet, these contributions are often small (Hubbard & Kamran, 2010), due to the desolvation penalty for disrupting water bonding in the unfolded state (Bolen & Rose, 2008). For the same reason, unsatisfied H bonds favor the unfolded state (McDonald & Thornton, 1994). We therefore assess H bond changes in two metrics: the increase in internal protein–protein bonds and the decrease of unsatisfied H bonds. BoostMut estimates intramolecular binding energy by assessing angles and distances, assuming 25 kJ/mol for ideal interactions and scaling down based on deviations (Fleming & Rose, 2005; Hubbard & Kamran, 2010). Further refinements account for the effect of bifurcated H bonds (Feldblum & Arkin, 2014) and inclusion of the binding energy of water‐mediated H bonds (Petukhov et al., 1999) (Figure 1d). Unsatisfied H bonds are defined as all potential donor and acceptors (N, O, S, and their bound H atoms within the protein) without interactions.

Protein flexibility is another key stability marker, as structural fluctuations can expose the hydrophobic core and cause unfolding (Bommarius & Paye, 2013). Increased flexibility typically correlates negatively with stability (Kamerzell & Russell Middaugh, 2008; Karshikoff et al., 2015; Yu & Huang, 2014), an insight leveraged in previous thermostability designs (Yu & Huang, 2014). Accordingly, we use lower root mean square fluctuations (RMSF) of the protein backbone C*α* atoms during the MD as an indicator of stabilization. Separately, we examine sidechain flexibility, where an increase may indicate loss of favorable local interactions. To compare flexibility between different amino acids, we normalized RMSFs using a custom benchmark dataset of expected flexibility per amino acid and degree of surface exposure (Figure 1d). We generated this benchmark set by running MDs on a curated set of protein structures, obtained by filtering the protein data bank (PDB) for protein‐only structures with high resolution (≤1 Å) and a minimum size (>100 aa, to retain a well‐defined hydrophobic core). With this set of 77 proteins, we generated the distributions of expected RMSFs for each amino acid type binned into “exposed,” “partially buried,” or “fully buried” based on a relative solvent‐accessible surface area (SASA) of >20% of the total surface, 0%–20%, or 0%, respectively (Chen & Zhou, 2005; Zhang et al., 2009). To each of the resulting 60 benchmark distributions, we fitted a gaussian kernel density estimation, scaled to values between 0 and 1, and enforced a monotonically decreasing curve (Figure 1e, Supplementary Figure S1), to only penalize unusual flexibility, but not rigidity.

BoostMut further assesses hydrophobic exposure. As the main driver of protein folding, hydrophobic interactions promote core packing, while polar residues remain solvent‐exposed. Consequently, exposed hydrophobic residues often destabilize proteins (Qing et al., 2022). Although some studies report a prevalence of mutations introducing hydrophobic surface residues (Broom et al., 2020; Nisthal et al., 2019), hydrophobic exposure is generally undesirable due to reduced solubility and aggregation (Broom et al., 2017). BoostMut therefore penalizes increased hydrophobic exposure by summing the SASA of all C atoms and all H atoms bound to C, relative to values again obtained via a custom benchmark set, which compensates for polarization and other effects ignored in this simplified definition (Li et al., 2010). For the benchmark, we generated normalized and monotonically decreasing distributions, reflecting the typical surface exposure of each amino acid (Supplementary Figure S2).

Besides these main metrics, BoostMut includes additional minor checks. Since *α*‐helices rely on local interactions, the sequence‐structure relationship manifests stronger than for other secondary structures (Ismi et al., 2022). We implement this knowledge by assessing helix propensity—a scale assigning an energetic penalty to specific amino acids introduced into *α*‐helices (Pace & Scholtz, 1998). While most residues exhibit mild penalties (0–4 kJ/mol), proline is a major exception (13.22 kJ/mol), allowing BoostMut to prevent proline‐induced helix breakage (Woolfson & Williams, 1990). Another determinant of helix stability is helix‐capping motifs, which complete a helix termini's H‐bonding network, and whose disruption introduces destabilizing unsatisfied H bonds (Aurora & Rosee, 1998). As these effects may only emerge in extended simulations, BoostMut explicitly evaluates whether a mutation interferes with helix capping. Helix propensity is only checked for residues at least five positions from helix termini, ensuring the evaluation occurs independently from helix caps.

In addition to H bonds, proteins are also stabilized via ionic interactions between positively and negatively charged residues (Kumar & Nussinov, 1999; Panja et al., 2020). To prevent mutations from disrupting these salt bridges, BoostMut tracks their average number throughout the MD. As is common, we define a salt bridge as any occurrence of a negatively charged oxygen atom in aspartic or glutamic acids closer than 4 Å to a positively charged amine group in arginines, lysines, or histidines (Barlow & Thornton, 1983; Makhatadze et al., 2003).

We also consider disulfide bonds, which stabilize proteins via covalently linked cysteine residues, thus favoring the folded conformation by reducing unfolded‐state entropy (Betz, 1993; Dombkowski et al., 2014; Feige et al., 2018). While typically designed by specialized algorithms rather than general stability predictors (Dombkowski et al., 2014; Wijma et al., 2014), we implemented an identification step in the input structure to prevent their disruption.

BoostMut evaluates each of these metrics for the three selections, except for non‐fluctuating metrics (helix analysis and disulfide bonds), which are only returned for the entire protein context. All metrics are then reported as the difference between mutant and wild type, returning 0 for a mutation with neither detrimental nor beneficial effects. While the individual outputs may inform users on specific effects of a given mutation, the main output of BoostMut is a composite score for ranking mutations. To that end, each metric is normalized by dividing by the standard deviation within the set, yielding z‐score‐like values with the wild‐type score (0) as the reference. The sign is adjusted such that a positive score consistently indicates a stabilizing effect. If the primary stability predictor also outputs a numerical score, it may be included as an additional metric for the ranking after normalization. The final BoostMut score is simply the mean of all metrics and is used to rank the mutations.

A BoostMut‐containing pipeline then runs as follows: (1) a primary predictor selects an initial set of mutations via classification or based on a cutoff, (2) each selected mutant is simulated in (typically short) MD simulations, with length and number of replicates based on available resources, followed by (3) BoostMut scoring of the mutations, enabling users to select any number of top‐ranked mutations for experimental validation. The required benchmark data can be obtained from our implementation for trajectories up to 5× 1 ns for four common force fields: CHARMM27 (Mackerell Jr. et al., 2004), OPLS (Kaminski et al., 2001), AMBER (Wang et al., 2000), and YAMBER (Krieger et al., 2004). We also provide a command line utility to easily generate reference files for BoostMut from custom benchmark datasets for users who require longer timescales, other force fields, or prefer selecting a more defined (e.g., protein class‐specific) or larger benchmark dataset.

We distribute BoostMut as a pip‐installable Python package with a command line interface allowing customization of analyses, selections, and trajectory lengths via command‐line flags. The main output is a tabular file containing raw metric scores and composite rankings. We also developed a simple app hosted on https://fuerstlab.shinyapps.io/BoostMutUI/ for interactive exploration of the results (Figure 1f).

To assess BoostMut's effectiveness and establish optimal parameters, we evaluated its performance using data we had available from previous thermostability engineering projects applying the FRESCO workflow. As this protocol employs energy calculations, short MD simulations on mutants passing a −5 kJ/mol energy cutoff, and visual inspection ensuring structural feasibility and rigidity, the experimentally tested mutations from this set are highly pre‐selected. We obtained data from FRESCO campaigns that stabilized three biotechnologically relevant enzymes: limonene‐1,2‐epoxide hydrolase (LEH) (Wijma et al., 2018), alcohol dehydrogenase 99 (ADH) (Aalbers et al., 2020), and 5‐hydroxymethylfurfural oxidase (HMFO) (Martin et al., 2018), resulting in 1251 threshold‐passing point mutations, of which a total of 339 mutations were experimentally verified.

While the original FRESCO protocol that generated this dataset simulated 5 independently seeded replicates of 50 ps, we were curious to examine the impact of MD simulation length on prediction accuracy and re‐simulated the 106 threshold‐passing LEH mutations for 5× 1 ns each.

**FIGURE 2:**
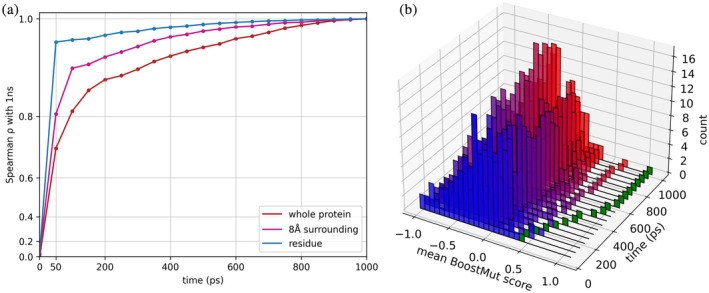
(a) Spearman *ρ* tracking the similarity of the BoostMut ranking on the LEH dataset to the final ranking at 1000 ps for each MD length, calculated for the three selections in intervals of 50 ps. Significantly higher Spearman *ρ* values for the residue and surrounding selections indicate faster convergence of the outcome compared to the whole protein selection. (b) Distribution of BoostMut scores for differing simulation times. The highly stabilizing mutation N92K (shown in green) is an example of a mutation benefitting from longer simulations times.

To track convergence of the ranking, we analyzed the Spearman ρ between variable trajectory lengths and the final 1 ns trajectory (Figure 2a). Noticing that the whole‐protein selection converges significantly more delayed (Figure 2a), we configured its use as an opt‐in setting in BoostMut, for example, for use cases where long MD (>500 ps) simulations are available.

As an exception, salt bridges are always analyzed on the whole protein, as they are less subject to noise due to their relative rarity, but are frequently absent at the residue or local level. While the overall correlation was already strong at 50 ps (*ρ* >0.8), some mutations showed a clear score change at longer simulation lengths, with the strongly stabilizing N92K mutation (Δ*T*_m_ + 7.3°C) becoming a noticeable outlier (Figure 2b). The higher score was primarily caused by this mutant's lysine sidechain adopting a distinct conformation that cemented subunit interaction, which led to a significantly reduced local flexibility as the simulation progressed (Supplementary Video 1).

To determine whether the choice of force field impacts the outcome, and whether it is imperative to use dedicated benchmarks, we re‐ran MDs with the same four force fields for which we generated the reference data. Upon comparison, we found that the final BoostMut score and mutation ranking are only marginally affected by force field, and that an analysis using a different benchmark has a minor practical influence on the selected mutations (Supplementary Figures S3–S6).

Since BoostMut analyses saved MD snapshots, we also examined the impact of trajectory output frequency. In the original data, a snapshot was saved every 5 ps, yielding 5× 10 files per mutation. We re‐ran the MDs with a tenfold higher sampling frequency and analyzed the convergence of the resulting mutation rankings (Supplementary Figure S7). Interestingly, while Spearman ρ reached ~0.9 at the 5 ps rate and full ranking convergence required sampling at least every 2.5 ps, BoostMut's performance did not improve beyond a snapshot interval of 25 ps. Lower sampling rates led to a steep drop in ρ and predictive performance. Consequently, we considered the original 5 ps interval adequate and used all resulting 5× 10 snapshots per mutant for further analysis.

We tested BoostMut on all 1251 mutations in our dataset, using data and benchmarks adjusted to the original protocol of 5× 50 ps per mutation (0.3 μs total simulation time). While the MDs correspond to a computational budget of a few days on a high‐end consumer GPU (Abraham et al., 2015; Eastman et al., 2017), BoostMut analysis of the combined 125,100 snapshots of all the mutations required approximately 60 total CPU hours, or ~3 minutes per mutation per CPU. The original visual inspections selected 339 mutations for experimental validation, forming our benchmark set. To avoid overestimating predictor performance, we did not use metrics like Pearson correlation, classification accuracy, or error rates (Diaz et al., 2024), which may let predictors that simply frequently classify mutations as destabilizing appear accurate. Instead, we used receiver operating characteristic (ROC) curves, which are independent of class distribution. The area under the ROC curve (AUC) quantifies ranking ability: 1 indicates perfect classification, 0.5 random sorting, and values below 0.5 reflect a preference for destabilizing mutations. To be deliberately conservative, we treated neutral mutations as unsuccessful, defining the classes as stabilizing and not stabilizing. As thermostability is a quantitative metric, we additionally propose a new metric, stability‐weighted ROC curves, where stabilizing mutations are scaled by their Δ*T*_m_ or ΔΔ*G* contribution. This approach rewards predictors that prioritize highly stabilizing mutations with higher AUCs, offering a metric of practical benefit in real‐world thermostability engineering.

**FIGURE 3:**
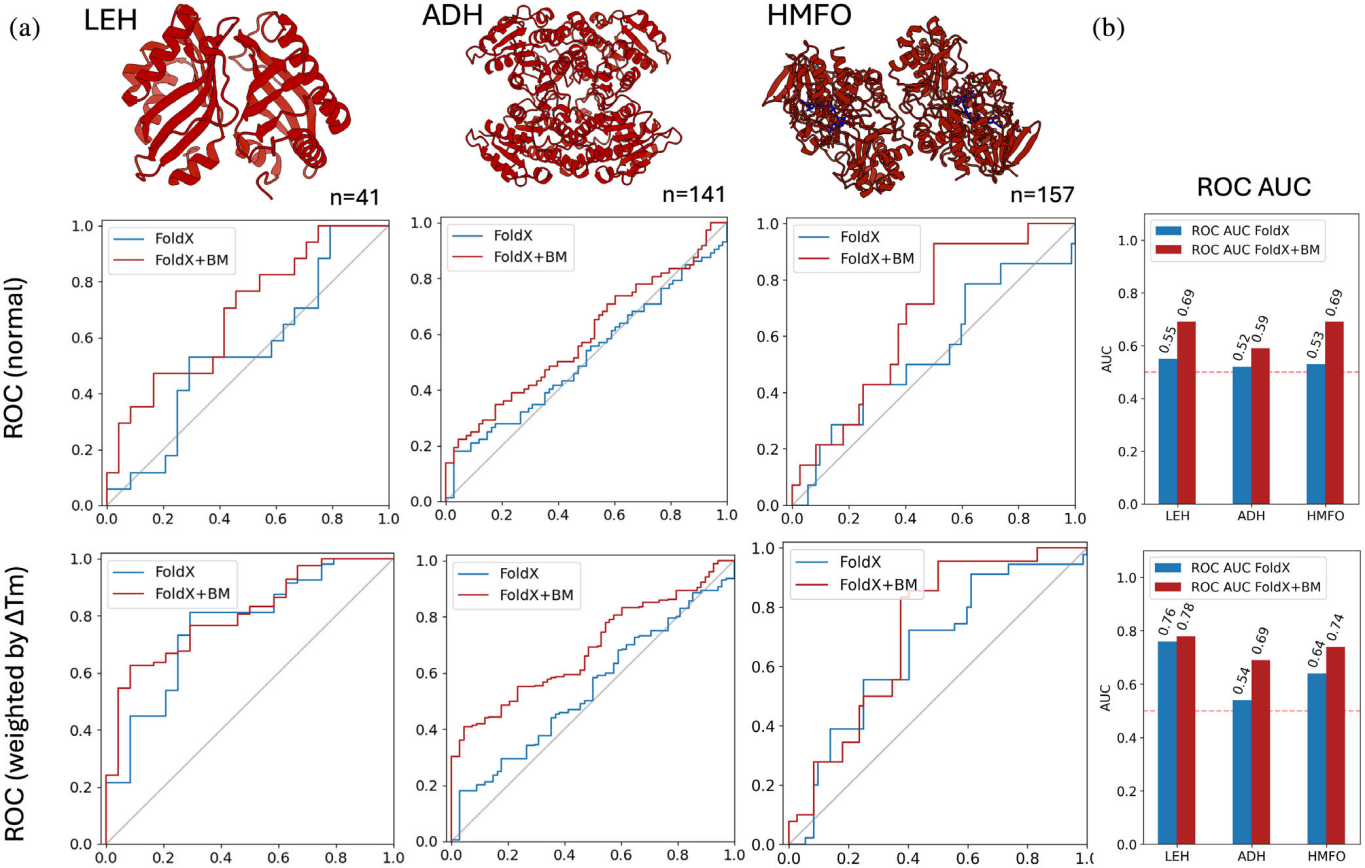
(a) Normal and weighted ROC curves for three proteins from previous stability engineering campaigns (LEH, ADH, HMFO) upon re‐ranking by BoostMut (BM). The weighted ROC curves scale each mutation by their respective Δ*T*_m_. (b) The improvements upon re‐ranking by BoostMut result in a consistent increase in AUC. The increase in AUC from the normal to weighted ROC curves indicates a preferential sorting of highly stabilizing mutations.

We first generated standard ROC curves for the FoldX scores of the 339 experimentally tested mutations. As expected, AUC values were low, only slightly above 0.5 (Figure 3b). This is unsurprising, as all mutations had not only been visually approved but also already passed a ΔΔ*G* cutoff of −5 kJ/mol—meaning FoldX already classified them as stabilizing, and its scores now correlate poorly within this narrow, pre‐selected range. This outcome reflects a broader limitation: predictors trained on datasets dominated by destabilizing mutations tend to perform poorly when used for ranking stabilizing ones. We then probed the outcome upon BoostMut filtering and found elevated AUC scores for all three proteins. Taken together, the average AUC score of all three proteins adjusted by sample size increases from 0.53 to 0.65 and from 0.61 to 0.72 for unweighted and weighted ROC curves, respectively. Notably, the stability‐weighted curves displayed higher AUCs regardless of ranking method (indicating that both FoldX and BoostMut prioritize highly over modestly stabilizing mutations), but BoostMut consistently ranked stabilizing mutations more effectively than FoldX alone. These results imply that BoostMut can increase both quantity and quality of stabilizing hits in a highly pre‐selected set, where primary predictors struggle to accurately distinguish mutation effects.

Since BoostMut functions as a secondary filter and requires MDs, we could not directly compare it to data from online databases (Diaz et al., 2024; Velecký et al., 2024). To expand the benchmark nonetheless, we evaluated the performance of BoostMut on a subset of the T2837 dataset used to benchmark Stability Oracle, a recent state‐of‐the‐art ML method (Diaz et al., 2024). This data comprises mutations with both positive and negative FoldX scores, allowing us to generate ROC curves that represent the combined effect of a complete BoostMut‐incorporating pipeline in which it ranks a primary algorithm's selection. We selected three proteins with over 100 mutations (PDB IDs 1BZ6, 1LZ1, 2LZM) and excluded those near cofactors, yielding 333 mutations. Since only a few passed the default thresholds, we used a relaxed cutoff of 0 kJ/mol, resulting in 107 pre‐selected mutations for FoldX and 102 for Stability Oracle. After recreating these mutants' structures with FoldX and running 5× 50 ps MD simulations, we ran BoostMut to rank them.

**FIGURE 4:**
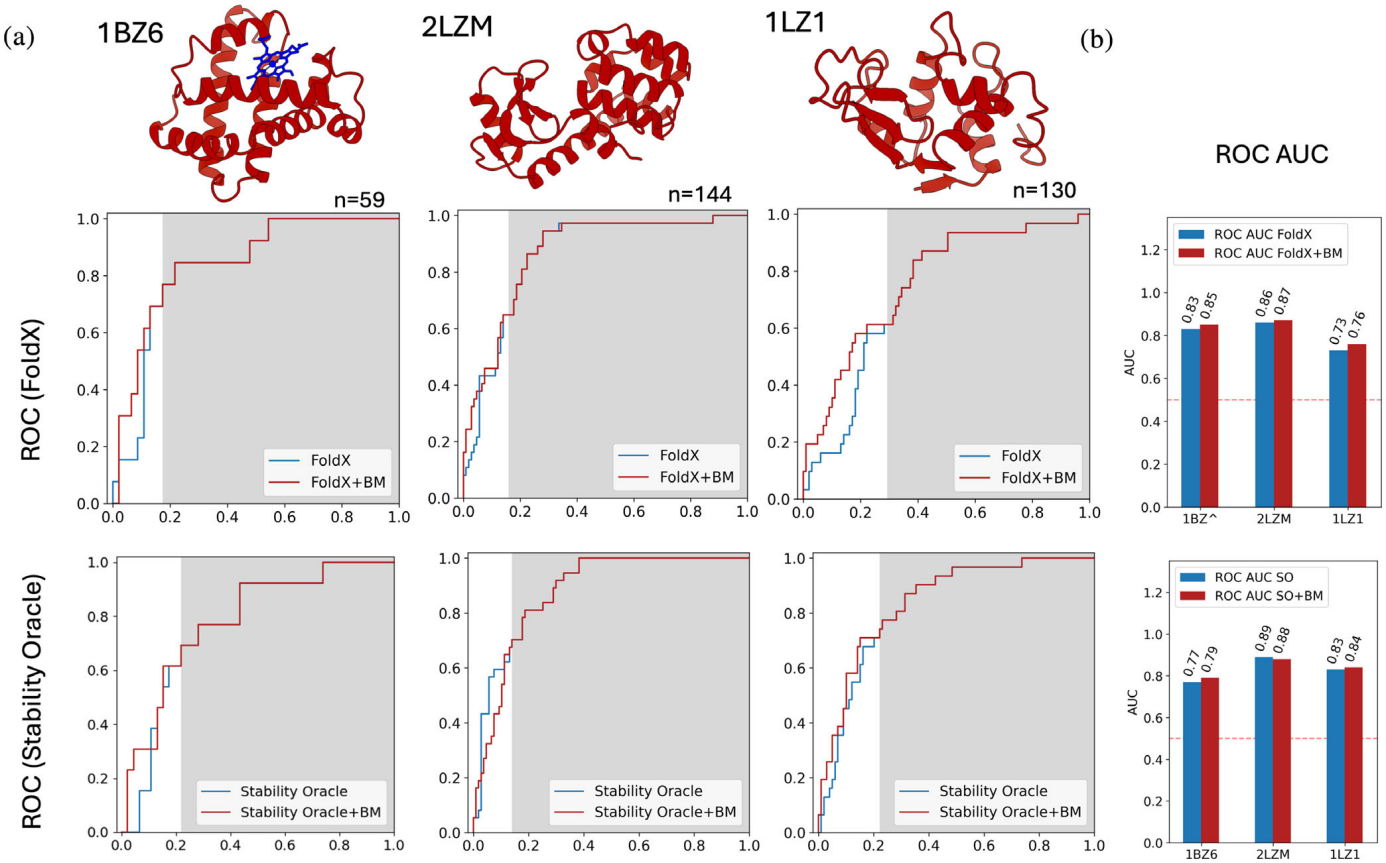
(a) ROC curves for three proteins from the T2837 test set, using either FoldX (top) or Stability Oracle (bottom) as the primary predictor. All mutations with a predicted ΔΔ*G* <0 were scored with BoostMut (white area), otherwise by the primary predictor (gray). (b) For 5/6 cases, the addition of BoostMut resulted in a small increase in ROC AUC.

In this analysis, the ROC curves led to significantly higher AUCs than for the FRESCO data, as one would expect with a significantly lower degree of preselection, which creates a more mixed set of mutations (Figure 4). In line with the intended BoostMut use, we reranked only the cutoff‐passing mutations (Figure 4a, white area), while the “rejected” mutations (Figure 4a, gray area) kept their original order. Here, the weighted ROC curves (Figure S8) showed a less pronounced difference, reflecting this set's narrower distribution of determined ΔΔ*G* values. In agreement with what was reported, we find Stability Oracle to slightly outperform FoldX on the whole: the average (sample‐size adjusted) AUCs were 0.84 and 0.80 for Stability Oracle and FoldX, respectively. Notably, the additional re‐ranking by BoostMut resulted in a small but consistent improvement, raising the average AUC from 0.80 to 0.82 for FoldX and raising the average AUC from 0.84 to 0.85 for Stability Oracle. Thus, BoostMut is apparently able to enhance the performance of diverse primary predictors and across different levels of preselection.

**FIGURE 5:**
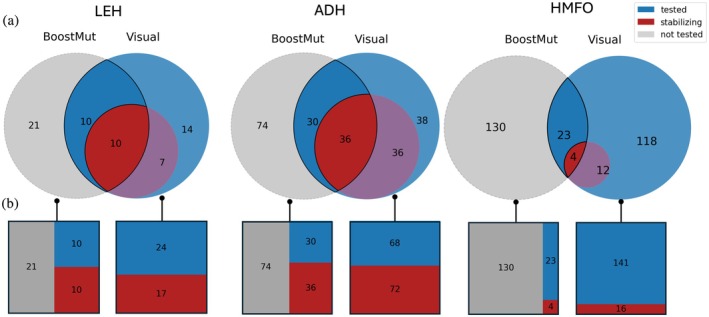
(a) Limited overlap in the mutations selected by visual inspection and BoostMut for three proteins from previous FRESCO campaigns (LEH, ADH, HMFO). (b) When comparing the mutations for which the experimental outcome is known, the fraction of stabilizing mutations is higher for BoostMut.

Since one of our key goals was replacing visual inspection for full automation in engineering pipelines, we directly compared BoostMut's performance to human filtering. Thus, we scored all 1251 MD‐simulated FRESCO mutations with BoostMut and selected the same number of top‐ranked mutations as were chosen manually: 41 for LEH, 141 for ADH, and 157 for HFMO. Notably, fewer than half of the two sets' mutations overlapped (Figure 5a). Yet, within the mutations selected by BoostMut with known Δ*T*_m_, stabilizing mutations were significantly enriched: 10/20 for LEH, 36/66 for ADH, and 4/27 for HMFO, at an average success rate of 50/103 or 49% (Figure 5b), outperforming visual inspection. However, as 225 of the BoostMut‐selected mutations and 912 of the remaining 1251 MD‐simulated FRESCO mutations lack experimental data, the true success rate remains unclear. Consequently, we chose to experimentally verify a feasible number of top‐ranked, uncharacterized mutations from LEH, where the smaller initial dataset would cause the new mutations to constitute a larger proportion of the whole. Among the 19 so‐selected mutations, Q7R, the highest‐ranked untested BoostMut mutation, had already been reported stabilizing by Sun et al., and we thus included its Δ*T*_m_ (Sun et al., 2023).

**FIGURE 6:**
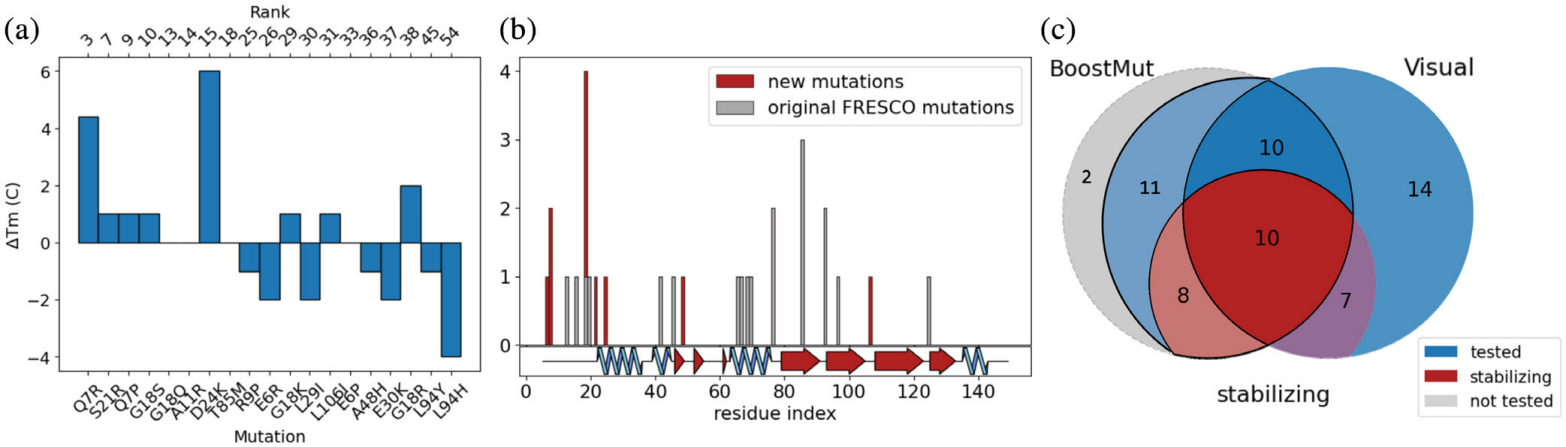
(a) Δ*T*_m_ of 19 the top ranked BoostMut mutations for LEH. (b) Location of all stabilizing mutations on the protein. Previously known mutations are gray, new ones red. (c) Venn diagram of the overlap between the mutations selected by visual inspection and by BoostMut when including the experimental data from the new mutations.

We generated *Escherichia coli* expression constructs for all new mutations via QuikChange of a pBAD expression vector for LEH, following established procedures (Wijma et al., 2014, 2018). All mutants were expressed with a 6xHis‐tag, purified by affinity chromatography, and desalted. Apparent melting temperatures were measured via the so‐called thermofluor assay (Pantoliano et al., 2001). Of the 19 newly selected mutations, 7 reduced *T*_m_, 4 were neutral, and 8 were stabilizing (Figure 6a), including 2 with Δ*T*_m_ increases >4°C—among the most stabilizing ever reported for LEH. Three mutations occurred at a previously identified stabilizing site, while five were at new positions (Figure 6b). Notably, the top four BoostMut‐ranked mutations were all stabilizing. Upon including the new mutations in the overall comparison, BoostMut's success rate for LEH increased further, with 18/39 (46%) stabilizing, compared to 17/41 (41%) for visual inspection (Figure 6c).

**FIGURE 7:**
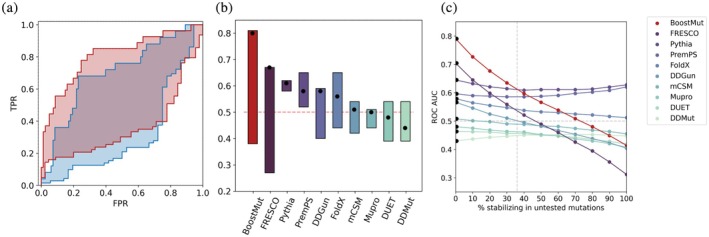
(a) Potential ROC curve ranges of visual inspection (blue) versus BoostMut (red), depicting the best and worst‐case scenarios for the remaining untested mutations. (b) Potential ranges of ROC AUC values for BoostMut, visual inspection, and other primary predictors. The scenario where all the remaining untested mutations are destabilizing is shown as black dots. (c) Changes in ROC AUC if an increasing fraction of untested mutations were actually stabilizing. BoostMut outcompetes all other methods until the scenario where 36% of untested mutations are stabilizing.

For a more thorough comparison, we again resorted to ROC analysis. However, despite the additional data, ROC analysis with all 108 cutoff‐passing LEH mutations remained complicated by the remaining 58/108 variants not selected for experimental verification by either method. To estimate possible outcomes, we programmatically evaluated all combinatorial scenarios, thus establishing upper and lower AUC bounds (Figure 7a). Since the original visual inspection followed a binary accept/reject approach, we ranked the mutations by FoldX scores, after placing all accepted before the rejected mutations. To compare more widely, we also evaluated this dataset with other structure‐based stability predictors. We thus applied Pythia (Sun et al., 2023), DDGun (Montanucci et al., 2022), DDMut (Zhou et al., 2023), DUET (Pires et al., 2014), Mupro (Cheng et al., 2006), mCSM, and PremPS (Chen et al., 2021) (Figure 7b) as an alternative means to score the mutations. Besides FoldX and Pythia, the BenchStab package was used to run the predictors (Velecký et al., 2024).

Our initial goal was to match human inspection performance, but BoostMut ultimately outperformed it. Across all possible outcomes for the untested mutations with unknown Δ*T*_m_, BoostMut scoring consistently resulted in higher AUC values than visual inspection, with bounds of 0.80–0.43 compared to 0.67–0.27, respectively (Figure 7). Regardless of whether the untested mutations would turn out to be all stabilizing or destabilizing, BoostMut retains an approximately 0.12 AUC advantage (Figure 7c). This indicates that BoostMut would likely outperform human judgment regardless of how many of the untested mutations would be identified as stabilizing. The large span in possible AUC values is caused by the low ranking of the remaining untested mutations rejected by both BoostMut and visual inspection—if any of these were in fact stabilizing, their position near the bottom of the ranking would strongly reduce the AUC. On the contrary, the other predictors are less sensitive to these fluctuations (Figure 7b), reflecting the fundamentally different algorithmic determination. However, we also found that all tested tools suffer from the same weakness in accurately predicting stabilizing compared to destabilizing mutations in this pre‐selected set, reinforcing the utility of secondary filtering. We find that as long as fewer than ~36% of untested mutations are stabilizing, BoostMut remains the best choice (Figure 7c). Given that all untested mutations were rejected by visual inspection, their expected stabilizing fraction should be at most 29%, based on the overall average success rate when using the default −5 kJ/mol FoldX cutoff (Buß et al., 2018). In our LEH, ADH, HMFO dataset, the fraction of stabilizing mutations was similar (31%), even after manual selection. If visual inspection actually served its intended purpose and led to an enrichment, this fraction would be significantly lower. If more than 36% of the remaining mutations were stabilizing, Pythia and PremPS—the two best‐performing predictors in our analysis—would outperform BoostMut. However, for this to occur, visual inspection would have needed to reject stabilizing mutations at a high rate, effectively lowering rather than enriching the success rate. Since this is unlikely, we expect the true stabilizing fraction to fall between 0 and 29%, rendering BoostMut the best choice for thermostable mutant filtering.

**FIGURE 8:**
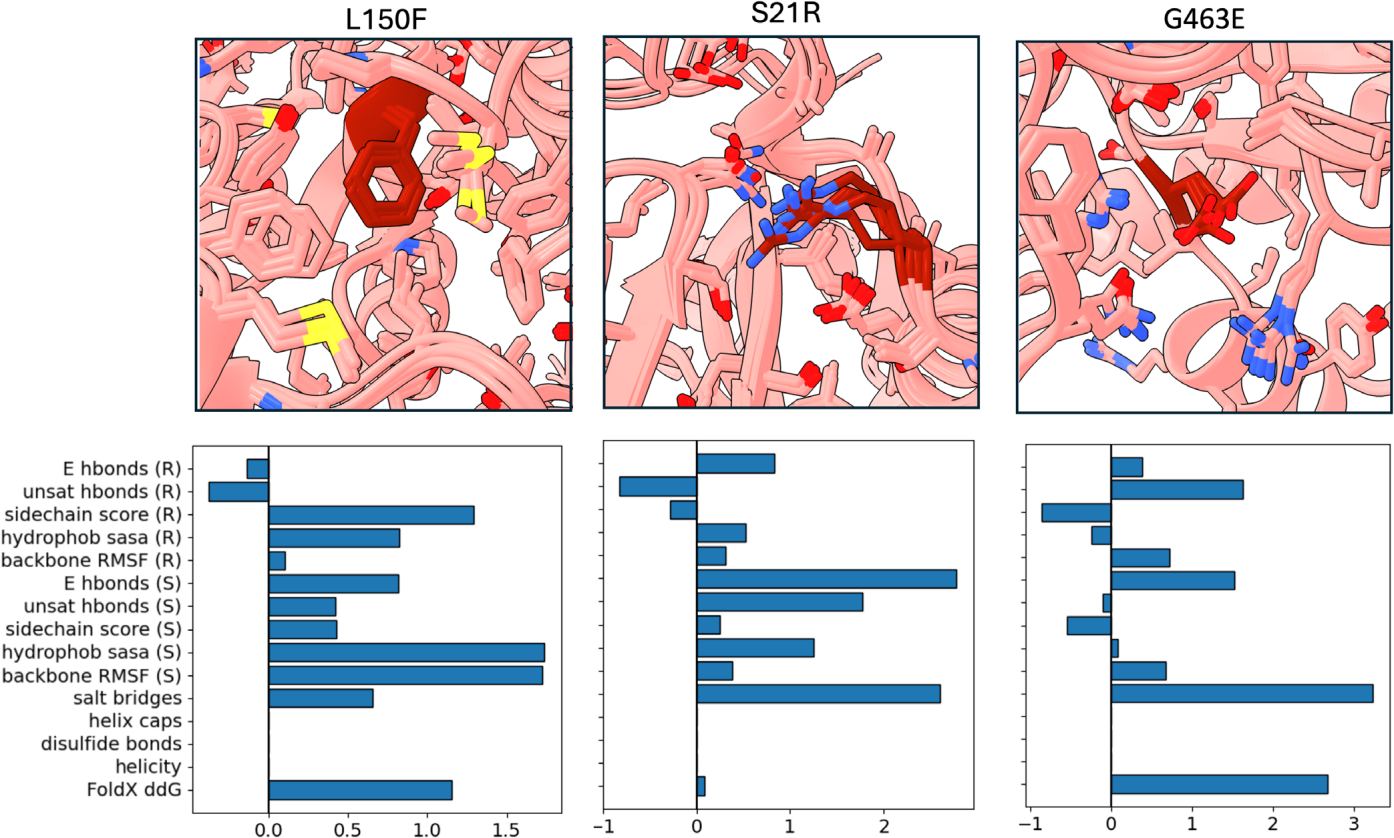
The scaled metrics for 3 selected mutations. L150F and S21R are both stabilizing mutations, successfully identified by BoostMut. G463E is a destabilizing mutation incorrectly classified as stabilizing by both visual inspection and BoostMut.

To better understand BoostMut's strengths and limitations, we examined illustrative examples of its performance (Figure 8). A notable success is L150F in ADH, which increased stability by 14.2°C. BoostMut scored it highly across several metrics, particularly for reduced hydrophobic exposure and lower backbone RMSF—consistent with visual inspection notes describing improved hydrophobic packing as the phenylalanine fills a cavity. S21R is a newly found mutation by BoostMut in LEH (+1°C Δ*T*_m_) that was manually rejected (Figure 6a). The main contributions to its positive BoostMut score come from the salt bridge and the H bond energy scores. While this improvement was also identified in the visual inspection, the mutation was rejected for a perceived increase in flexibility. However, both the backbone RMSF and the sidechain BoostMut scores are favorable, demonstrating the value of a more objective metric in evaluating flexibility. Other examples also highlight limitations of MD‐based screening. For instance, G463E in HMFO was favored by both visual inspection and BoostMut due to apparent improvement in hydrogen bonding and salt bridge formation, but the mutation was experimentally found to decrease stability. To systematically compare BoostMut and visual inspection, we manually examined all 43 mutations in LEH where their selections differed. In most cases (29/43), the underlying observations were largely in agreement, but differences in prioritization led to divergent outcomes. The remaining 14 cases involved direct conflicts, primarily in flexibility or hydrogen bonding, both of which are challenging to assess visually.

**FIGURE 9:**
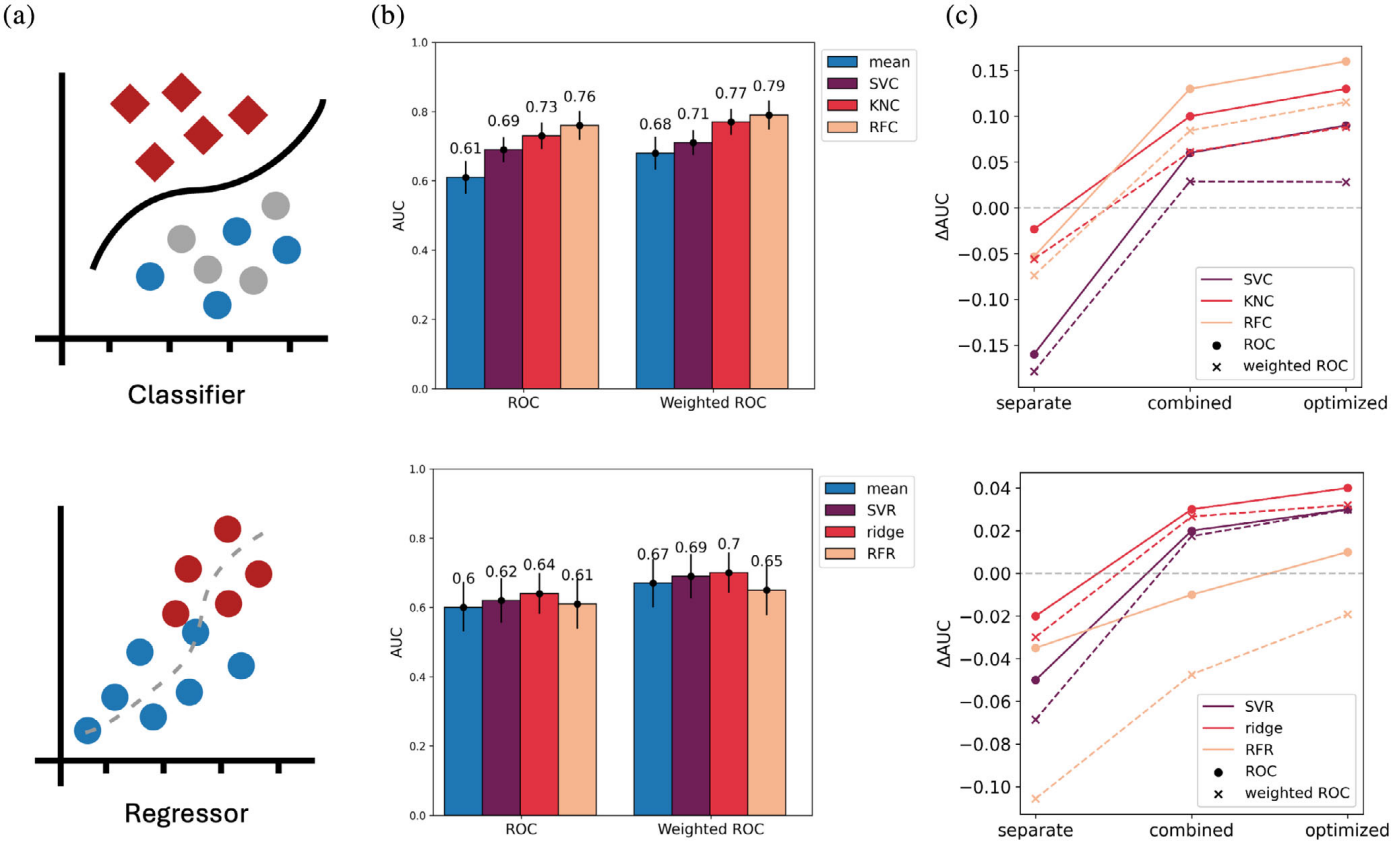
(a) The machine learning task can be trained to classify known stabilizing mutations from the set of all mutations (*n* = 1251), or as a regressor predicting the Δ*T*_m_ from the set of experimentally tested mutations (*n* = 216). (b) Weighted and unweighted ROC AUC values for three classifiers: a support vector classifier (SVC), a K‐nearest cluster classifier (KNC), and a random forest classifier (RFC). Similarly, three regressor models were tested: a support vector regressor (SVR), a ridge regressor (ridge), and a random forest regressor (RFR). The random forest classifier shows the largest increase in AUC for the classifier task, and the ridge regression for the regression task, respectively. (c) Optimization of the model training: the models were trained either separately on each of the proteins, trained on data from all the proteins combined, or with all proteins combined + optimized hyperparameters using a grid search. The difference in AUC between the simple mean and the predictor is shown (ΔAUC).

We reasoned that BoostMut's consistent assessment of biophysical stabilization mechanisms may provide a useful dataset for machine learning. While BoostMut by default simply averages all metrics to a final score, we asked whether we could improve performance by training a model on BoostMut's output. Although fully realizing this potential would require a larger‐scale analysis, we tested six common ML estimators from the scikit‐learn library in Python (Pedregosa et al., 2011) on the FRESCO mutations as a proof of concept. Predicting stabilizing mutations can be framed as either a classification (stabilizing or not) or a regression task (predicting Δ*T*_m_) (Figure 9a). For this reason, we tested three classifiers—a support vector classifier (SVC), K‐nearest neighbors classifier (KNC), and a random forest ensemble classifier—and three regressors—a support vector regressor (SVR), ridge regressor, and random forest ensemble regressor (RFR). Random forest models were chosen as representative ensemble methods due to their robustness and minimal tuning requirements (Parmar et al., 2019). We trained the classifiers on all mutations (*n* = 1251), effectively training the model to pick stabilizing mutations that would also be chosen by visual inspection. For the regressors, only the mutations where the Δ*T*_m_ was experimentally confirmed (*n* = 216) were used, meaning the model task became the prediction of Δ*T*_m_ on the mutations pre‐selected by visual inspection.

We performed repeated stratified k‐fold cross validation using 4 splits and 10 repeats. To assess whether the ML models could improve performance, we plotted the averaged weighted and unweighted ROC curves for all six estimators (Figure 9b), ranking by model probability and Δ*T*_m_ for classifier and regressor, respectively.

We tried either training the models on each of the proteins separately or combining data across all proteins and investigated the effect of optimizing hyperparameters (Figure 9c). The results showed that the combined data resulted in a significant improvement in both weighted and unweighted AUC for all classifiers and regressors, while hyperparameter tuning resulted in a modest increase in AUC. Notably, the regressors only marginally increased AUCs compared to the simple mean, possibly due to the too‐small amount of training data. Conversely, the classifiers led to a significant AUC improvement, with the random forest model leading to the highest increase in both AUC and weighted AUC. This performance boost suggests that once a sufficient number of mutations are identified, training a lightweight model can significantly increase the success rate and demonstrates BoostMut's utility for ML.

## 3 DISCUSSION

We developed BoostMut to improve the identification of stabilizing mutations and assist in prioritizing individual mutations for experimental validation, complementing any primary predictor. This effort was motivated by the observation that while existing algorithms effectively filter out destabilizing mutations, their correlation with stabilizing effects is relatively weak (Khan & Vihinen, 2010). BoostMut refines pre‐selected mutation sets by providing a more reliable ranking than a single primary predictor would achieve.

The concept of reranking mutation sets is not entirely new. BoostMut builds on pre‐existing thermostability pipelines by formalizing the visual inspection of MD simulations previously employed in protocols such as FRESCO (Wijma et al., 2018) and GRAPE (Sun et al., 2024), allowing for the incorporation of dynamic information into otherwise purely structure‐based predictions. Similar multi‐step filtering approaches are also common in pipelines such as FireProt (Musil et al., 2017) or HotSpot Wizard (Sumbalova et al., 2018), where evolutionary rather than dynamic information is integrated as additional filters. Other approaches have attempted to integrate multiple tools into one meta‐predictor (Broom et al., 2017).

BoostMut's role as a secondary filter presented a key benchmarking challenge, as it operates on mutation sets already enriched for stability, complicating comparisons with other tools. Consequently, we relied on mutations already enriched for stability, potentially introducing biases in the datasets used for evaluation. We here used ROC curves to account for the imbalance between stabilizing and destabilizing mutations and propose weighted ROC analysis to better reflect stability improvements. While ROC curve weighting has precedent, it is commonly implemented as weights to classes or ROC curve sections rather than individual data points (Li & Fine, 2010; Wang & Li, 2012).

To evaluate BoostMut's performance and additionally account for biases in individual datasets, we used four complementary approaches: (1) We re‐ranked 320 FRESCO‐proposed mutations and found an improvement of ROC AUC on a mutation set where the high degree of pre‐selection caused the primary predictor's score to perform poorly. (2) To further benchmark BoostMut on a dataset not pre‐selected for stability, we tested 333 mutations from the T2837 dataset, which included a more diverse set of mutations, and showed that BoostMut improved AUC when used alongside both the empirical force‐field‐employing FoldX and the recent ML predictor Stability Oracle. (3) We compared BoostMut to visual inspection on 1251 MD‐simulated FRESCO mutations, where it selected significantly different mutations, but achieved a higher success rate (46%) among the overlapping mutations. (4) We experimentally validated 19 BoostMut‐selected LEH mutations, of which 8 were stabilizing, demonstrating that BoostMut enhances selection even in cases where mutations were rejected manually. Across all tests, BoostMut consistently improved performance as a secondary filter.

Based on these results, we believe that BoostMut can replace visual inspections in thermostability engineering when time or expertise is limited, or augment it with objective guidance. Designed for broad compatibility, BoostMut remains independent of the primary stability predictor, allowing flexibility as methods, in particular ML‐based, evolve. Beyond filtering, BoostMut may also enhance AI‐driven thermostability predictions: its biophysical approach provides an orthogonal check on black‐box ML models, helping identify prediction inconsistencies that would be physically obvious. Moreover, its machine‐readable, standardized output can serve as feedback for further training, potentially aiding in the development of biophysics‐aware ML algorithms. We take a step in this direction by demonstrating that an ML model tasked with optimizing metric weighting can be trained on BoostMut output.

Another strength of BoostMut is its implementation flexibility. While we show that longer MDs improve success rates, we find that the ranking converges relatively quickly and leave this choice to the user, and even support static structure input. Similarly, users can customize metric priorities, facilitating system‐specific considerations. For example, a salt bridge design campaign may warrant BoostMut use with other metrics disabled, while highly soluble proteins might justify deprioritizing hydrophobic exposure.

Although BoostMut can be paired with any thermostability prediction method, the reliance on MD simulations adds a computational burden that may limit how many mutations can practically be assessed. Consequently, MD‐based screening approaches such as FRESCO are typically restricted to short MD simulations, and we have adopted the default 5× 50 ps timescales in our analysis for consistency. Yet, for smaller sets of mutations—or if computational resources permit—BoostMut can be implemented with longer simulations. Although we expect system‐specific behavior in absolute terms, our data suggest that the benefits from extended MDs up to 5× 1 ns are modest. Remaining misclassifications may stem from limitations in energy calculations or mutant structure predictions, but also from dynamic effects such as molecular interactions and conformational changes. The latter may only become detectable at significantly extended timescales, in which case enabling BoostMut's whole‐protein selection—which we observed to converge more slowly—could prove advantageous.

In summary, BoostMut provides a powerful tool for MD‐supported secondary stability mutation filtering. Although MD simulations add a computational burden, the improvement in accuracy typically outweighs the additional cost and labor of experimentally assessing a larger fraction of false positive mutations. With ongoing developments in conformational ensemble generation, such short MD simulations could in the future be replaced by generative ML models with a much lower computational cost (Jing et al., 2024; Lewis et al., 2025). Besides ranking, BoostMut metrics may also serve as labels for large mutation datasets in the future training of improved ML models.

## 4 MATERIALS AND METHODS

BoostMut is implemented as a python package with a command line program called boostmut_run. All analyses are performed on 3 selections: the whole protein, the local surrounding of the mutation, and just the interactions with the mutation itself. For the local surrounding, we use a default cutoff radius of 8 Å, which has been suggested as an optimal context to assess effects on stability (Gromiha et al., 2000). A second command line interface called boostmut_process provides tools to process the raw output, such as adding primary predictors as metrics, scaling the values by standard deviation, or outputting the data into a human‐readable excel format. An online tool implemented as an R shiny app for the visualization of the metrics and data is freely available at https://fuerstlab.shinyapps.io/BoostMutUI/.

### 4.1 Hydrogen bonding

The analysis of both the H bond energy and unsaturated H bonds is calculated and averaged over each frame in the trajectory. H bonds are identified using the hbond_analysis function in the MDAnalysis package. The donor‐hydrogen‐acceptor angle and donor‐acceptor distance cutoffs used to identify H bonds are set to be more lenient than the default settings (an angle cutoff of 100° instead of the default 150° and distance cutoff of 3.5 Å instead of the default 3 Å respectively) as later steps filter out unrealistic H bonds using an approximation of their bonding energy.

The bonding energy is approximated based on the angles and distances of the H bond, allowing BoostMut to work without charge information. The energy function is inspired by the ListHBo command in YASARA, which we further modified. The formula scales down an ideal interaction energy of 25 kJ/mol based on deviations in the distance (Scale_HA_) and angle (Scale_DHA_) away from the ideal conformation (Hubbard & Kamran, 2010). Energy is further scaled down if any atom covalently bound to the acceptor is close enough to the hydrogen to hinder the H bond (Scale_HAX_). The formula as provided by YASARA is then as follows:

$$E_{\mathrm{hbo}}=25*{\text{Scale}}_{\mathrm{HA}}*{\text{Scale}}_{\mathrm{DHA}}*{\text{Scale}}_{\mathrm{HAX}}$$

The formulas for each of the scaling factors are saturated ramp functions that scale between 0 and 1. The ramp function of Scale_HAX_ depends on whether the covalent atom X is a hydrogen or a heavy atom. Since the latter causes more steric hindrance, stricter criteria are applied. If there are multiple covalent atoms attached to the acceptor, the lowest scaling factor is used.

$${\text{Scale}}_{\mathrm{HA}}=\frac{2.6-\max\left({\text{Dist}}_{\mathrm{HA}},2.1\right)}{\left(2.6-2.1\right)}$$

$${\text{Scale}}_{\mathrm{DHA}}=\frac{\min\left({\text{Angle}}_{\mathrm{DHA}},165\right)-100}{\left(165-100\right)}$$

$$\begin{array}{l} {\text{Scale}}_{\mathrm{HAX}}=\frac{\min\left({\text{Angle}}_{\mathrm{HAX}},95\right)-85}{\left(95-85\right)}\text{or}\\ \frac{\min\left({\text{Angle}}_{\mathrm{HAH}},85\right)-75}{\left(85-75\right)} \end{array}$$

For bifurcated H bonds, the second bond is scaled down to a maximum of 15 kJ/mol (Feldblum & Arkin, 2014). The formula was applied to all H bonds identified by MDAnalysis, and only H bonds with an energy higher than 6.25 kJ/mol are selected.

First order Water‐mediated H bonds or water bridges are included in the H bond energy (Petukhov et al., 1999). Water bridges are identified by first selecting all waters involved in two or more H bonds with the protein and then selecting all protein‐water H bonds containing these waters. The bond energy of a water bridge is estimated by summing the energies of all H bonds involved in the water bridge and subtracting an entropic penalty of 32.2 kJ/mol for removing a water from solution (Petukhov et al., 1999). To find the number of unsatisfied hydrogen bonds, the set of all atoms engaged in an H bond is compared to the set of all potential H bond partners (defined as all oxygen atoms and all nitrogen atoms not bonded to any hydrogens). Whenever an atom is found in the set of potential H bond partners, but not in the set of atoms engaged in a hydrogen bond, the atom is counted as an unsatisfied hydrogen bond.

### 4.2 Protein flexibility

Both the RMSF of the backbone and the flexibility score of the sidechain are calculated. The flexibility of the backbone is measured by calculating the RMSF of the Cα atoms in the backbone using the MDAnalysis package. For the RMSF values of the sidechains, a local alignment of ±1 amino acid around the residue is performed for each residue so that the RMSF score is not dominated by fluctuations in the backbone, and the RMSF for the sidechain atoms excluding the backbone is calculated.

For the sidechain score, benchmark distributions were categorized based on amino acid type and whether the residue was buried, partially exposed, or fully exposed, resulting in a total of 20 × 3 = 60 different benchmark distributions (Figure S3). The three categories were defined using the fraction of solvent‐exposed surface (SASA), calculated with freeSASA (Mitternacht, 2016; Shrake & Rupley, 1973). The residue is considered buried if 0% of the residue is solvent exposed, partially exposed if between 0% and 20% is solvent exposed, and fully exposed if the residue is >20% solvent exposed (Chen & Zhou, 2005; Kumar & Nussinov, 1999; Miller et al., 1987). The percentage of exposure is defined as the SASA value divided by the average SASA of the single amino acid in solution reported in the literature (Durnham et al., 2009).

To create the benchmark curves, a Gaussian kernel density estimation (KDE) was fitted to the distributions for each category obtained from the benchmark data set (described below), with the bandwidth set using Silverman's rule of thumb. For each of the 60 categories, the maximum value was scaled to 1, allowing for a mapping from the sidechain RMSF to a sidechain score between 0 and 1. In order to guarantee the score improves monotonically when the sidechain RMSF is lowered, each value was set to the maximum between either its own value or the highest score among all RMSF values higher than the current one (i.e., “filling in” the local minima). The resulting curves are saved as a series of *x* and *y* values in .csv format for each of the categories.

### 4.3 Hydrophobic surface exposure

The degree of hydrophobic surface exposure is determined by calculating SASA with FreeSASA and averaged over the trajectory. Hydrophobic moieties were approximated as all atoms engaged in a C–C or C–H bond. A set of curves with distributions of hydrophobic surface exposure per amino acid was created from the benchmark dataset, resulting in 20 categories. The benchmark curves were processed identically to the RMSF benchmark curves: A Gaussian KDE was fitted to the distributions for each category obtained from the test set, with the bandwidth set using Silverman's rule of thumb. The maximum value was scaled to 1, and the local minima were flattened to guarantee a monotonic decrease.

### 4.4 Other metrics

Besides H bonds, flexibility and surface exposure, four other structural metrics are recorded which could disrupt protein stability. All *α*‐helices are identified on the first frame of the simulation using pyDSSP (Kabsch & Sander, 1983). To find helix capping motifs, all possible motifs are checked within 5 amino acids around the start or end of the helix to account for fluctuations in the assignment of the secondary structure (Yang et al., 2016). To track helix propensity, we take the scale reported in Pace et al. and track its change for any residues inside an *⍺*‐helix and 5 residues away from the start or end of the helix (Pace & Scholtz, 1998). To verify that no cysteine‐cysteine disulfide bonds have been disrupted by any of the mutations, we count the number of bonds between cysteine residues before and after mutation. The number of salt bridges is counted by taking all cases where an atom capable of being an electron donor is less than 4 Å away from an atom capable of being an electron acceptor (Kumar & Nussinov, 1999). Only the number of salt bridges is averaged over each frame of the trajectory and reported for all three selections.

### 4.5 High throughput molecular dynamics

High throughput MD were performed using either GROMACS (for AMBER99 (Wang et al., 2000), CHARMM27 (Mackerell Jr. et al., 2004), or OPLS force fields (Kaminski et al., 2001)) or the YASARA software package (for the YAMBER3 force field (Krieger et al., 2004)), using the same protocol previously reported for FRESCO (Wijma et al., 2018). This pipeline involves the following steps: addition and optimization of hydrogens using the YASARA software, solvation and neutralization of the simulation cell with 0.5% NaCl, followed by a steepest descent minimization. The system is then brought up to a temperature of 298 K in a 20 ps equilibration run, followed by a 50 ps production run. Unless otherwise stated, a snapshot was taken every 5 ps. For the FRESCO mutations, all simulated steps (minimization and MD) were done using the Amber‐derived YAMBER3 force field, with long range interactions calculated using the particle mesh Ewald (PME) method. For the benchmark distributions, simulations were run for the YAMBER3, AMBER99, CHARMM27, and OPLS force fields.

For further analysis by BoostMut, the YASARA‐specific .sim files were converted to a format recognized by MDAnalysis. The .sim files were converted into a .xtc file using the md_convert macro provided by YASARA. Besides the trajectory, a topology file containing at least atom and bond information should be provided for the automated inspection, which can either be done by using GROMACS to generate a .trp from a given .pdb, or by explicitly saving bond information into the pdb.

For the three proteins with more than 100 mutations (1BZ6, 2LZM, 1LZ1) reported in the T2837 test set, the structures for all mutations were generated using FoldX and run using the high‐throughput MDs. For LEH, the original MD trajectories from Floor et al. were preserved. For ADH and HMFO, MD simulations were re‐run using the same initial structures and simulation parameters as the original FRESCO campaign.

### 4.6 Benchmark data set

A benchmark set of proteins was obtained from the PDB. In order to obtain well‐behaved, representative proteins, all proteins were selected with (1) a refinement resolution of <= 1 Å, (2) a length of 100 amino acids or larger, and (3) no non‐proteogenic entities present: (total number of non‐polymer entities = 0, polymer entity type = protein). This resulted in 77 proteins with a length distribution between 100 and 501 amino acids.

The same pipeline for high‐throughput MD as described above were run on each of the structures for 1000 ps. To get the data for the RMSF benchmark curves, each residue in each of the proteins in the test set was categorized based on surface exposure and amino acid type, and its RMSF recorded. For hydrophobic exposure, each residue was categorized based on amino acid type and its hydrophobic surface exposure was recorded. Benchmarks were generated for simulation times between 50 and 1000 ps in 50 ps intervals by slicing the 1000 ps trajectory to the desired length.

### 4.7 Experimental characterization of new mutations

A synthetic gene encoding LEH was ordered from Integrated DNA Technologies after BsaI sites were added to the 5′‐and 3′‐termini. The gene encoding LEH was cloned into a pBAD His vector using golden gate cloning (Engler & Marillonnet, 2014). To further facilitate automation, we created a streamlined python implementation of AAscan, a software package used to design partially overlapping mutagenesis primers previously used in FRESCO and other high‐throughput protein engineering campaigns (Wijma et al., 2018). The python implementation (https://github.com/kt-korbeld/pyAAscan) (Sun et al., 2013) is based on the original AAscan code (https://github.com/dbv123w/AAScan). Primers for the mutations were designed to be overlapping, and each mutation was introduced using the QuikChange protocol utilizing PfuUltra Hotstart PCR Master Mix II. The product was transformed into NEB10*β* cells, and all mutations were verified through direct colony sequencing using a ColonySeq plate provided by Eurofins. Each mutation was expressed by inoculating an overnight culture of the transformed NEB10*β c*olonies into 50 mL of TB containing 50 μg/mL ampicillin. Once the cultures reached an OD_600_ of 0.6–1.0, they were induced with 0.02% L‐arabinose and incubated for 16 h at 30°C. The cells were harvested by centrifugation at 15,000 × *g* for 1 h at 4°C and stored at −20°C until needed.

During purification, the buffers used were those reported in the most recent engineering campaign on LEH (Wijma et al., 2015). To purify the protein, the pellets were resuspended in lysis buffer (50 mM Hepes, 500 mM NaCl, pH 8) until they reached an OD_600_ of 12, lysed using sonication (5 min total time, 5 s on, 10 s off), and the cell debris was removed by centrifugation (15,000 G, 1 h, 4°C). The cell‐free extract was loaded onto an equilibrated Ni‐Sepharose column and left to incubate for 1 h at 4°C. The column was washed with washing buffer (50 mM Hepes, 500 mM NaCl, 20 mM imidasol, pH 8) for 10 column volumes (CV), after which the protein was eluted with 5 CVs of elution buffer (50 mM Hepes, 500 mM NaCl, 300 mM imidasol, pH 8). The obtained protein was desalted using a desalting column, obtaining the final protein in a desalted buffer (50 mM Hepes, pH 8).

To measure the melting temperature, 20 μL each of the isolated protein variants (with a concentration between 0.5 and 2.5 mg/mL) was loaded into transparent PCR tubes together with 5 μL of 100‐fold diluted Sypro Orange (Life Technologies, CA, USA), after which the plate was sealed with transparent foil. The fluorescence (excitation at 490 nm and emission at 575 nm) was monitored while the sample was heated from 20 to 99°C at 1.1°C/min in a RT‐PCR machine (CFX96 Touch Real‐Time PCR, BioRad, signal setting: FRET). The melting temperature was obtained by taking the maximum of the first derivative of the fluorescence curve over time.

### 4.8 ROC AUC analysis

Normal ROC AUC is plotted using the sklearn.metrics.roc_curve function. For the weighted ROC AUC, we implement a custom version of the ROC function that weighs each change in the true positive rate by the relative contribution to the total increase in Δ*T*_m_. As we are not interested in the degree a mutation is destabilizing, the true negatives are not scaled.

To identify the best and worst‐case scenarios among the remaining untested mutations, we calculate the upper and lower bounds of the ROC AUC. We ignore all permutations where the best and worst‐case scenarios do not have all true positives sorted as close as possible to either the highest or lowest rank, respectively. We therefore iteratively change each untested mutation to be stabilizing from highest to worst ranked and from worst to highest ranked, calculating the AUC for each scenario.

We also plot the ROC AUC as a function of an increasing fraction of stabilizing mutations within the untested mutations. Here we are no longer interested in a particular best‐ or worst‐case scenario for the ROC AUC, but calculate the average effect of a given fraction of positive hits, independent of its exact sorting. To calculate this for each fraction, the desired number of positive hits is randomly introduced among the unknown mutations and the new ROC AUC is calculated. This is repeated 1000 times to average out the effect of any particular sorting.

### 4.9 Machine learning

The scikit.learn implementation of each classifier and regressor was used. The support vector machines were implemented with sklearn.svm.SVC and sklearn.svm.SVR. The KNC was implemented using sklearn.neighbors.KNeighborsClassifier, and the ridge regressor using sklearn.linear_model.Ridge. The Random Forest approaches were implemented using sklearn.ensemble.RandomForestClassifier and sklearn.ensemble.RandomForestRegressor. For both classifiers and regressors, the scaled BoostMut metrics were provided as features. For classifier models, all 1251 simulated mutations were used, and classes of stabilizing or destabilizing mutations were provided as labels. For the regressor models, only the 216 experimentally verified mutations were used, and the Δ*T*_m_ values were provided as labels. The models were cross‐validated using repeated stratified k‐fold cross‐validation, using 4 splits and 10 repeats, and the averaged ROC curves and AUC values are reported. For the classifiers, the model probabilities were used as predictions to generate the ROC curves. Models were either trained on each of the proteins separately (in which case the average AUC and weighted AUC across the three proteins are reported) or trained on the mutations from all proteins combined. Hyperparameters were optimized using a grid search, optimizing for the highest average of both the weighted and unweighted AUC.

### 4.10 Other analyses and visualization

All figures were produced using the matplotlib and seaborn python libraries. Venn diagrams were generated using matplotlib‐venn. Protein structures were visualized using YASARA or ChimeraX. The secondary structure representation in Figure 6 was generated using the secstructartist python package. Predictions using DDGun (Montanucci et al., 2022), DDMut (Zhou et al., 2023), DUET (Pires et al., 2014), Mupro (Cheng et al., 2006), mCSM, and PremPS (Chen et al., 2021) were generated using the Benchstab command line interface (Velecký et al., 2024). Predictions for FoldX were calculated using protocols established for the FRESCO pipeline (Wijma et al., 2018). Predictions for Pythia (Sun et al., 2023) were generated using the colab notebook provided at https://colab.research.google.com/gist/JinyuanSun/83ff4323ff751dc665f96381a02df18a/colabpythia.ipynb#scrollTo=iJ58Cx4z2lof.

## AUTHOR CONTRIBUTIONS

**Kerlen T. Korbeld:** Investigation; writing – original draft; methodology; validation; visualization; software; formal analysis; data curation. **Maximilian J. L. J. Fürst:** Conceptualization; funding acquisition; writing – original draft; methodology; validation; writing – review and editing; project administration; supervision; resources.

## CONFLICT OF INTEREST STATEMENT

The authors declare no conflicts of interest.

## Supporting information


**Figure S1.** Benchmark curves for expected sidechain flexibility (RMSF) for each of the different force fields at 50 ps simulation time, for 60 categories, resulting from classification of each amino acid into “exposed” (>20% relative SASA), “partial” (>0% and <20% relative SASA), and “buried” (0% relative SASA). Distributions of RMSF for each of the categories are obtained from the benchmark simulations with the respective force field, fitted with a Gaussian kernel, scaled to a maximum value of 1, and made to decrease monotonically.
**Figure S2.** Benchmark curves for expected hydrophobic exposure (SASA) per amino acid for each of the different force fields at 50 ps simulation time. Distributions of hydrophobic SASA for each of the categories are obtained from the benchmark data, fitted with a Gaussian kernel, scaled to a maximum value of 1, and made to decrease monotonically. The benchmark curves are relatively invariant to changes in force field.
**Figure S3.** The difference in BoostMut score when using different RMSF benchmark curves on all simulated ADH mutations (*n* = 397). A high Spearman *ρ* (~0.95) indicates that the ranking of mutations remains largely unaffected.
**Figure S4.** The overlap in the selections of the top 100 mutations ranked by BoostMut score when using different RMSF benchmark curves on all simulated ADH mutations (*n* = 397). In all cases the overlap remains around 80%–90%.
**Figure S5.** The difference in BoostMut score when using different SASA benchmark curves on all simulated ADH mutations (*n* = 397). A very high Spearman *ρ* (~0.99) indicates that the ranking of mutations remains unaffected.
**Figure S6.** The overlap in the selections of the top 100 mutations ranked by BoostMut score when using different SASA benchmark curves on all simulated ADH mutations (*n* = 397). In all cases the overlap remains around 96%–98%.
**Figure S7.** (a) Spearman *ρ* between variable output frequencies of the number of frames saved to a trajectory and an output frequency of 0.5 ps for all simulated mutations of ADH (*n* = 397). The gray dotted line indicates the default FRESCO sampling rate (5000 fs/frame). (b) The effect of a lower sampling rate on the average weighted and unweighted ROC AUC for all experimentally tested mutations across all three proteins (*n* = 216). Especially the weighted AUC is affected at lower sampling rates, indicating the ranking of highly stabilizing mutations benefits from more dynamic data.
**Figure S8.** (a) Weighted ROC curves for three proteins from the T2837 test set, using either FoldX (top row) or Stability Oracle (bottom row) as the primary predictor. All mutations with a predicted ΔΔ*G* <0 were scored with the BoostMut (the area shown in white), all mutations with a predicted ΔΔ*G* >0 were scored by the primary predictor (the area shown in gray). (b) For all cases apart from Stability Oracle when used on 2LZM, the addition BoostMut resulted in a small increase in the weighted ROC AUC.

**Video 1:** Supporting Information

## Data Availability

Source code, instructions for BoostMut, and all data and analysis used to generate the figures are available at https://github.com/kt-korbeld/BoostMut. The MD trajectories associated with the study are available upon request.
