# Supplementary material for: Enriching stabilizing mutations through automated analysis of molecular dynamics simulations using BoostMut
Source: Protein Sci. 2025 Oct 11;34(11):e70334. doi: 10.1002/pro.70334 (PMC12514941; doi:10.1002/pro.70334)
Supplement: Supplementary file 1 — Figure S1. Benchmark curves for expected sidechain flexibility (RMSF) for each of the different force fields at 50 ps simulation time, for 60 categories, resulting from classification of each amino acid into “exposed” (>20% relative SASA), “partial” (>0% and <20% relative SASA), and “buried” (0% relative SASA). Distributions of RMSF for each of the categories are obtained from the benchmark simulations with the respective force field, fitted with a Gaussian kernel, scaled to a maximum value of 1, and made to decrease monotonically. Figure S2. Benchmark curves for expected hydrophobic exposure (SASA) per amino acid for each of the different force fields at 50 ps simulation time. Distributions of hydrophobic SASA for each of the categories are obtained from the benchmark data, fitted with a Gaussian kernel, scaled to a maximum value of 1, and made to decrease monotonically. The benchmark curves are relatively invariant to changes in force field. Figure S3. The difference in BoostMut score when using different RMSF benchmark curves on all simulated ADH mutations (n = 397). A high Spearman ρ (~0.95) indicates that the ranking of mutations remains largely unaffected. Figure S4. The overlap in the selections of the top 100 mutations ranked by BoostMut score when using different RMSF benchmark curves on all simulated ADH mutations (n = 397). In all cases the overlap remains around 80%–90%. Figure S5. The difference in BoostMut score when using different SASA benchmark curves on all simulated ADH mutations (n = 397). A very high Spearman ρ (~0.99) indicates that the ranking of mutations remains unaffected. Figure S6. The overlap in the selections of the top 100 mutations ranked by BoostMut score when using different SASA benchmark curves on all simulated ADH mutations (n = 397). In all cases the overlap remains around 96%–98%. Figure S7. (a) Spearman ρ between variable output frequencies of the number of frames saved to a trajectory and an output frequency of 0.5 ps for all [file PRO-34-e70334-s002.docx]

Supplementary Materials for:

Kerlen T. Korbeld^1^, Maximilian J.L.J Fürst^1*^

^1^Molecular Enzymology, University of Groningen, The Netherlands

*Corresponding author, email: [m.j.l.j.furst@rug.nl](mailto:m.j.l.j.furst@rug.nl)

Table of Contents

[Figure S1 2](#_Toc201395273)

[Figure S2 3](#_Toc201395274)

[Figure S3 4](#_Toc201395275)

[Figure S4 4](#_Toc201395276)

[Figure S5 5](#_Toc201395277)

[Figure S6 5](#_Toc201395278)

[Figure S7 6](#_Toc201395279)

[Figure S8 7](#_Toc201395280)

## Figure S1


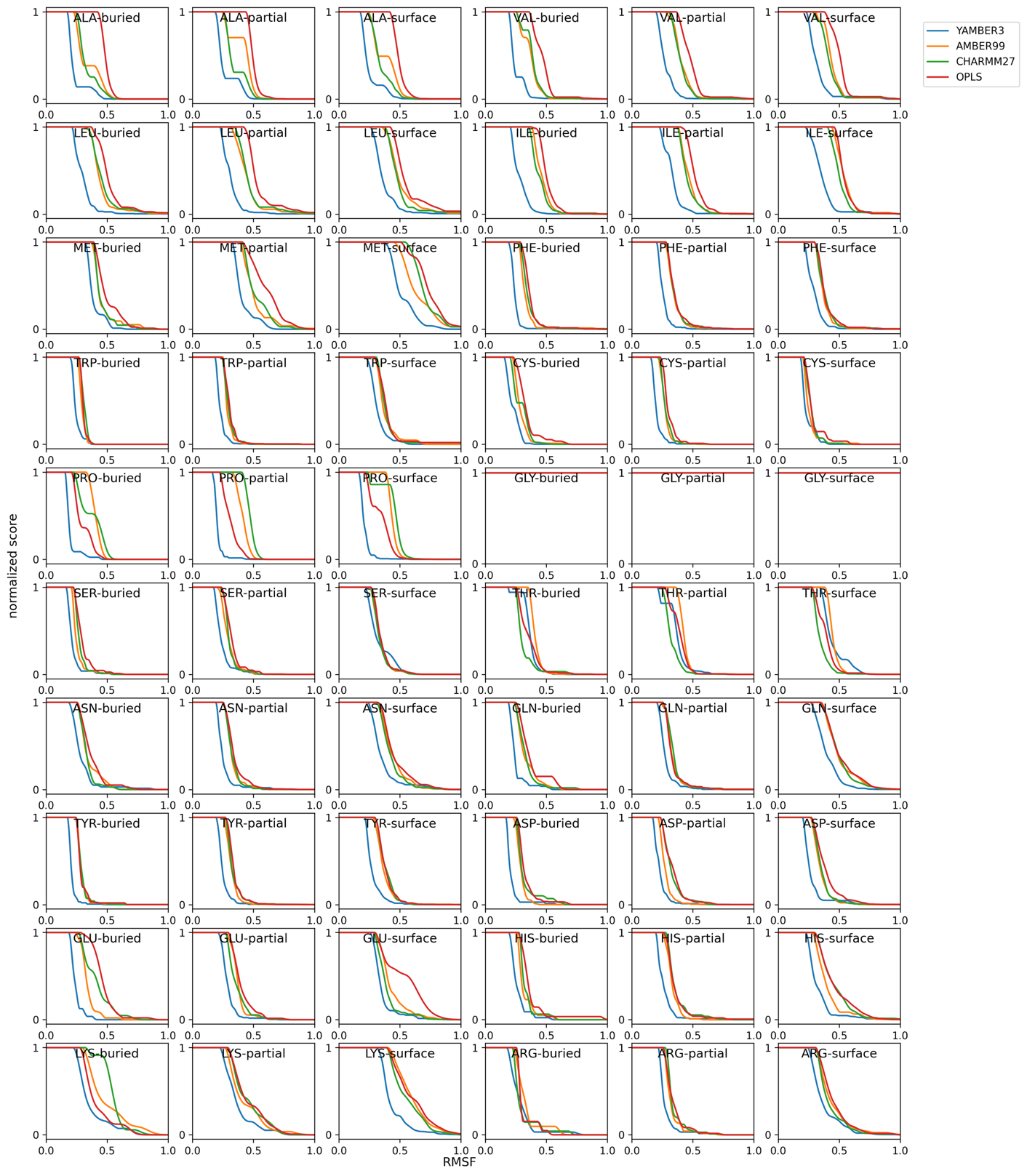


Figure S1 Benchmark curves for expected sidechain flexibility (RMSF) for each of the different forcefields at 50 ps simulation time, for 60 categories, resulting from classification of each amino acid into “exposed” (>20% relative SASA), “partial” (>0% and <20% relative SASA), and “buried” (0% relative SASA). Distributions of RMSF for each of the categories are obtained from the benchmark simulations with the respective force field, fitted with a Gaussian kernel, scaled to a maximum value of 1, and made to decrease monotonically.

## Figure S2


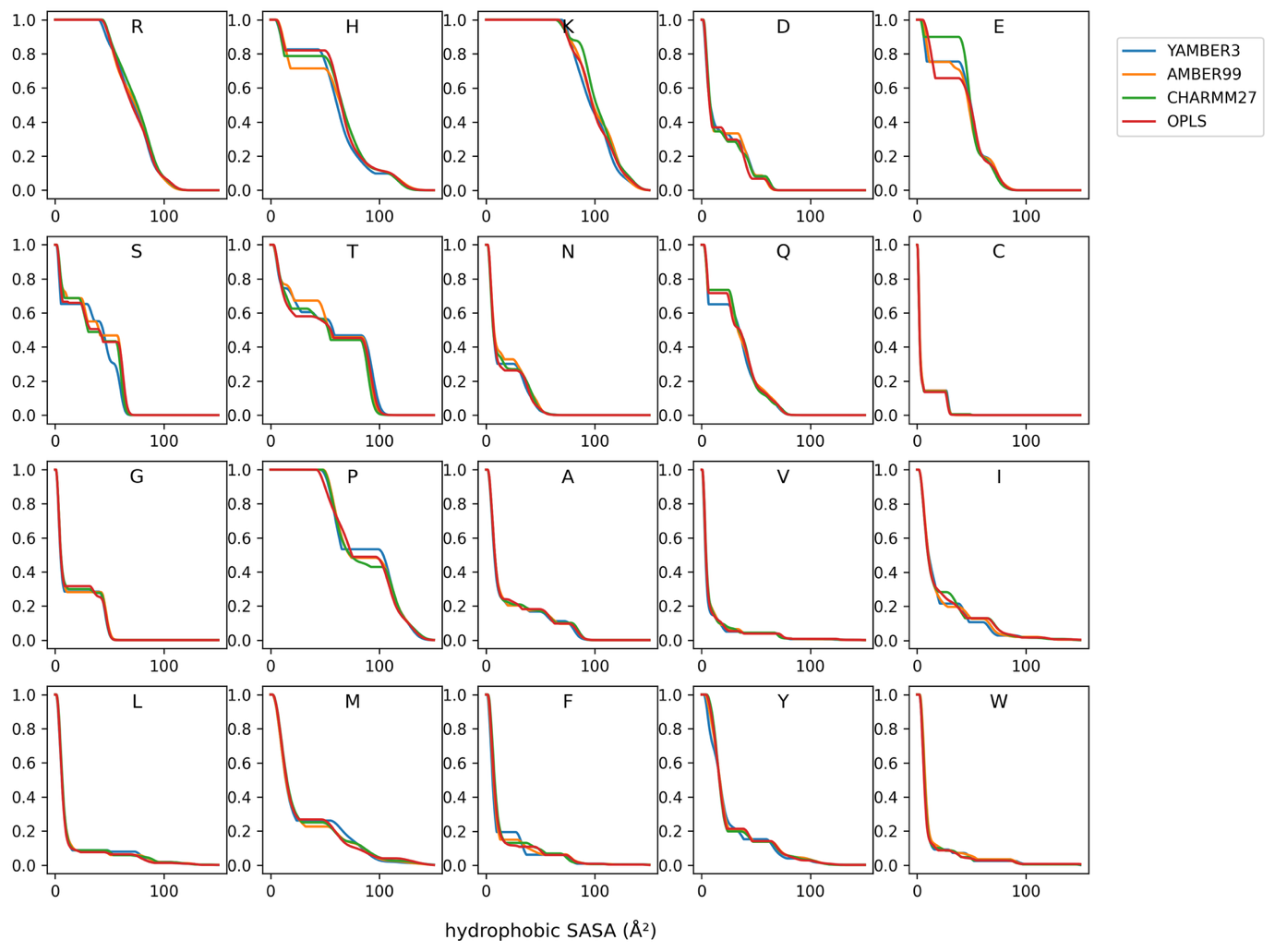


Figure S2 Benchmark curves for expected hydrophobic exposure (SASA) per amino acid for each of the different forcefields at 50 ps simulation time. Distributions of hydrophobic SASA for each of the categories are obtained from the benchmark data, fitted with a Gaussian kernel, scaled to a maximum value of 1, and made to decrease monotonically. The benchmark curves are relatively invariant to changes in forcefield.

## Figure S3


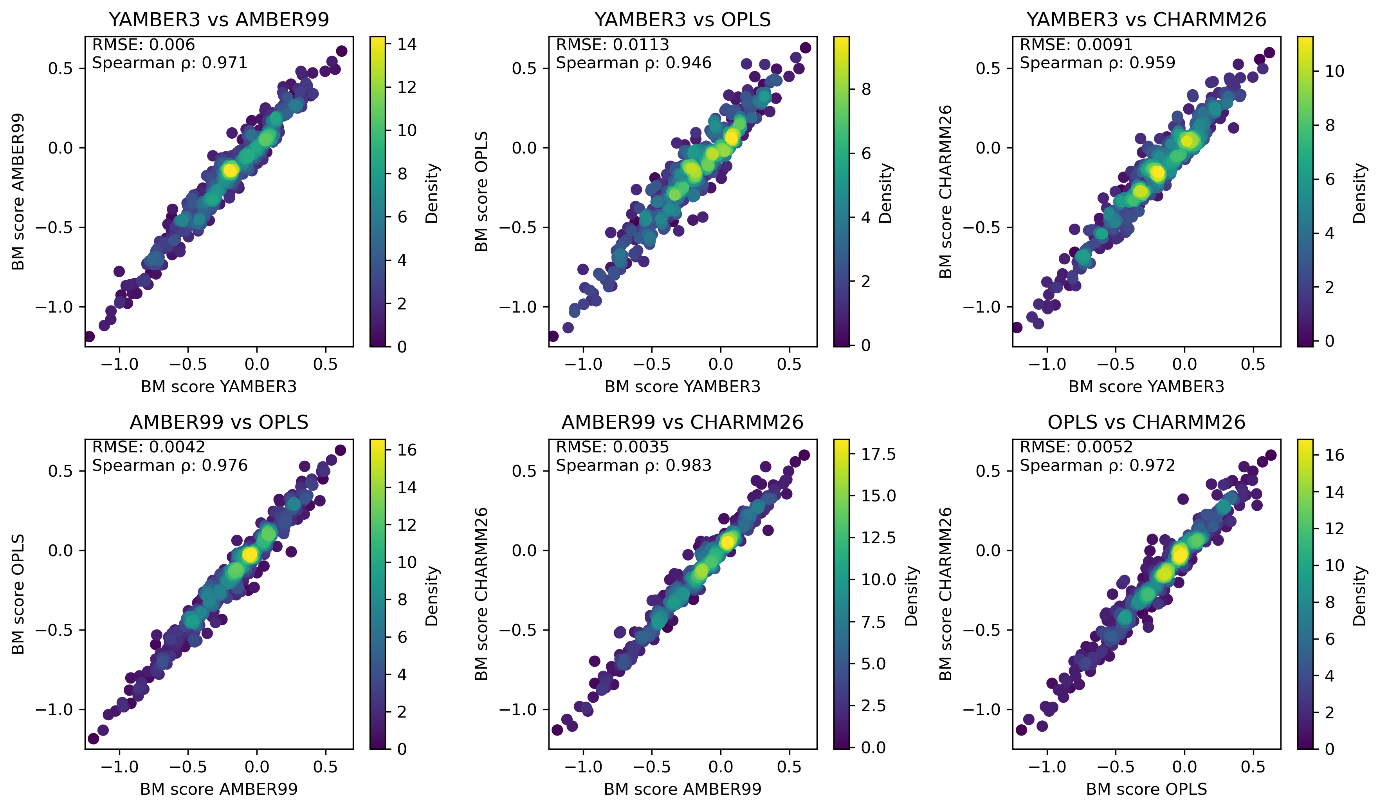


Figure S3 The difference in BoostMut score when using different RMSF benchmark curves on all simulated ADH mutations (n=397). A high Spearman ρ (~0.95) indicates that the ranking of mutations remains largely unaffected.

## Figure S4


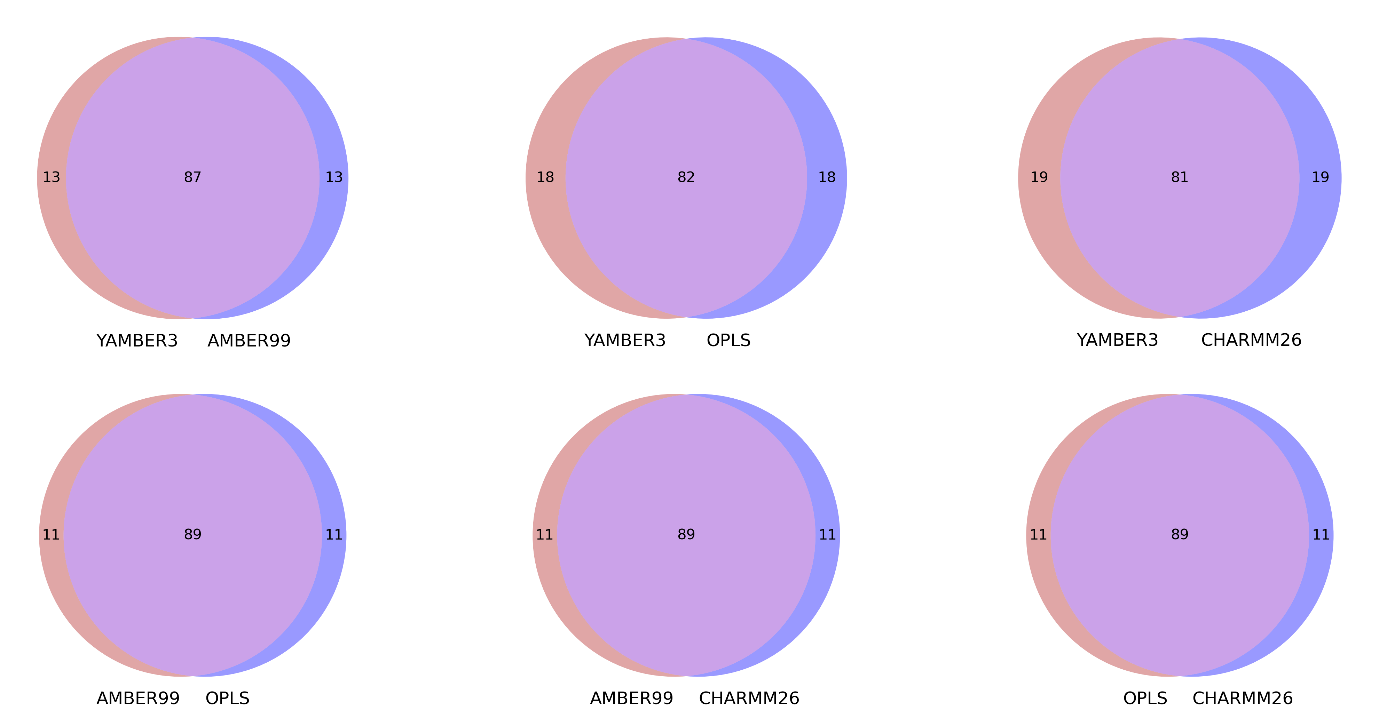


Figure S4 The overlap in the selections of the top 100 mutations ranked by BoostMut score when using different RMSF benchmark curves on all simulated ADH mutations (n=397). In all cases the overlap remains around 80-90%

## Figure S5


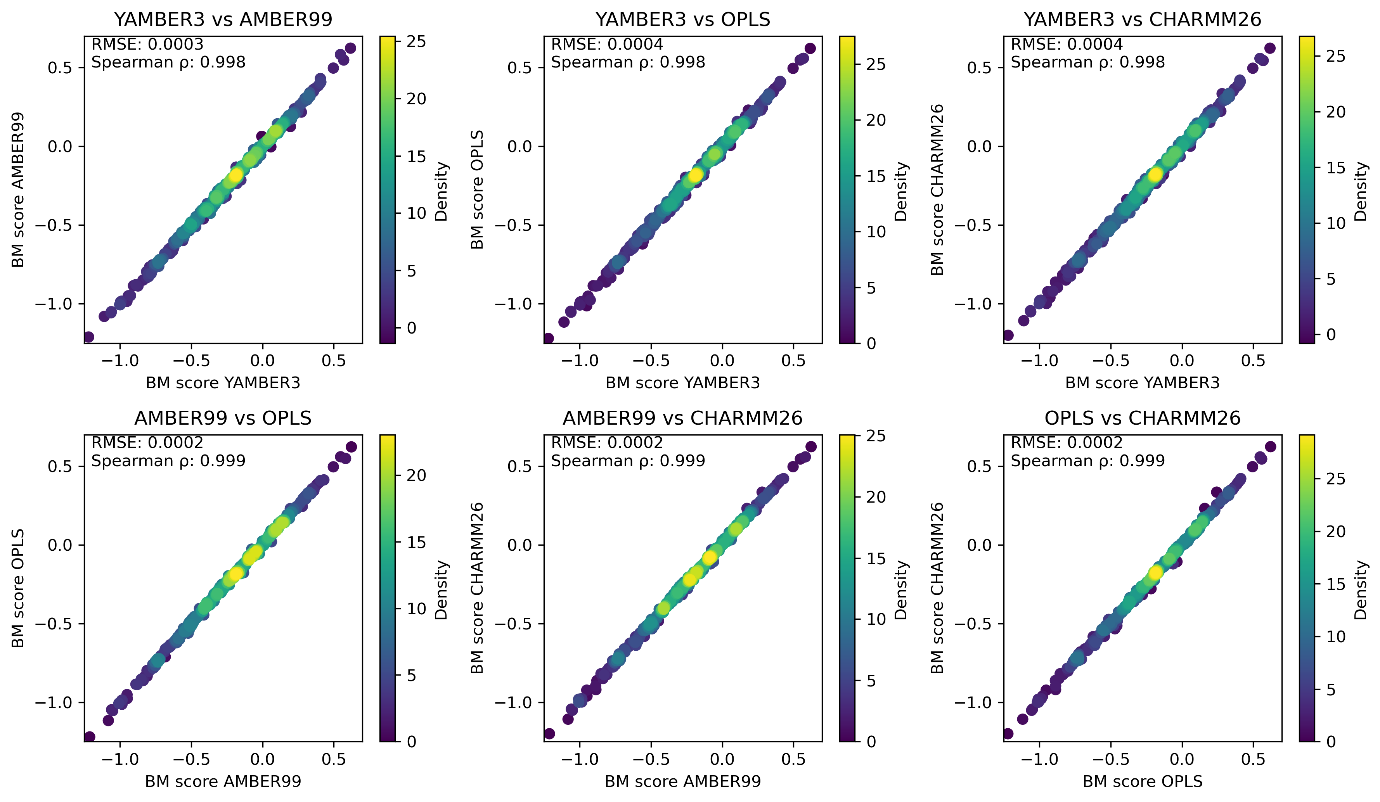


Figure S5 The difference in BoostMut score when using different SASA benchmark curves on all simulated ADH mutations (n=397). A very high Spearman ρ (~0.99) indicates that the ranking of mutations remains unaffected.

## Figure S6


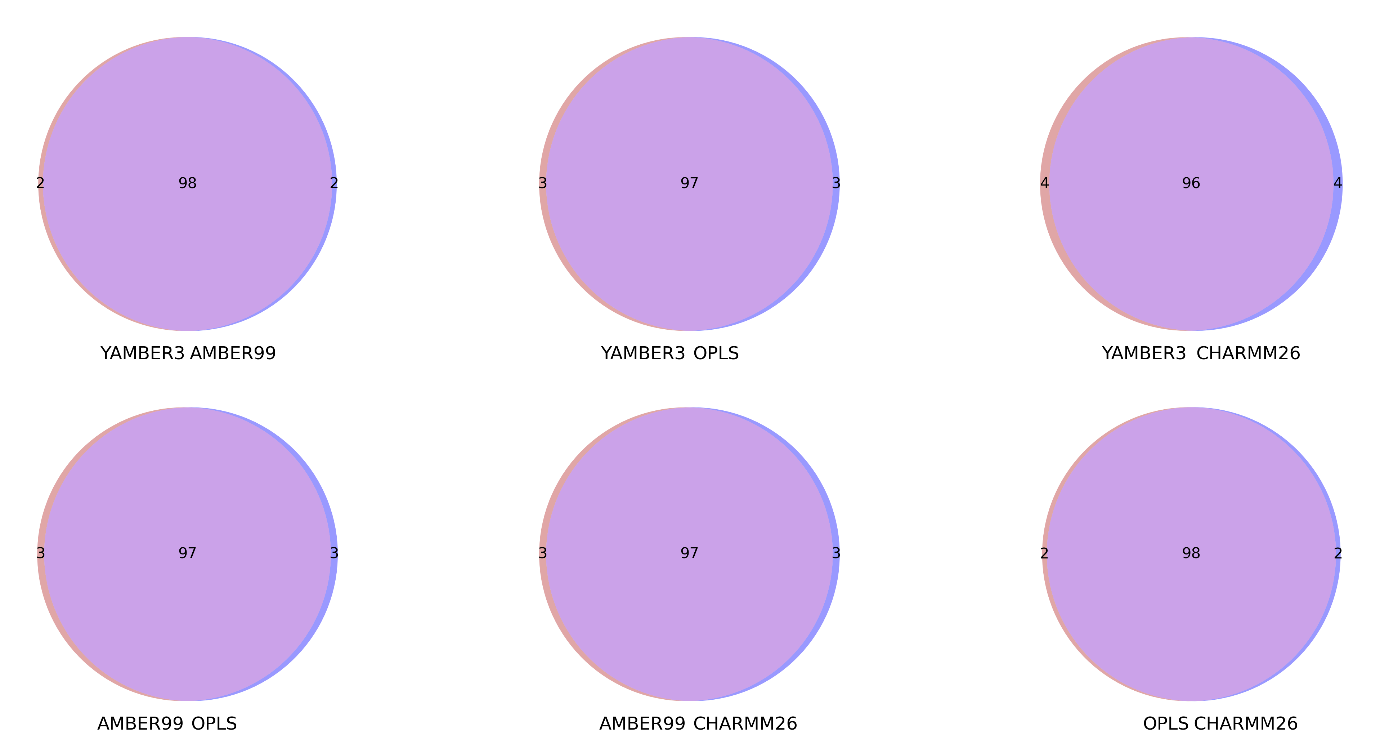


Figure S6 The overlap in the selections of the top 100 mutations ranked by BoostMut score when using different SASA benchmark curves on all simulated ADH mutations (n=397). In all cases the overlap remains around 96-98%

## Figure S7


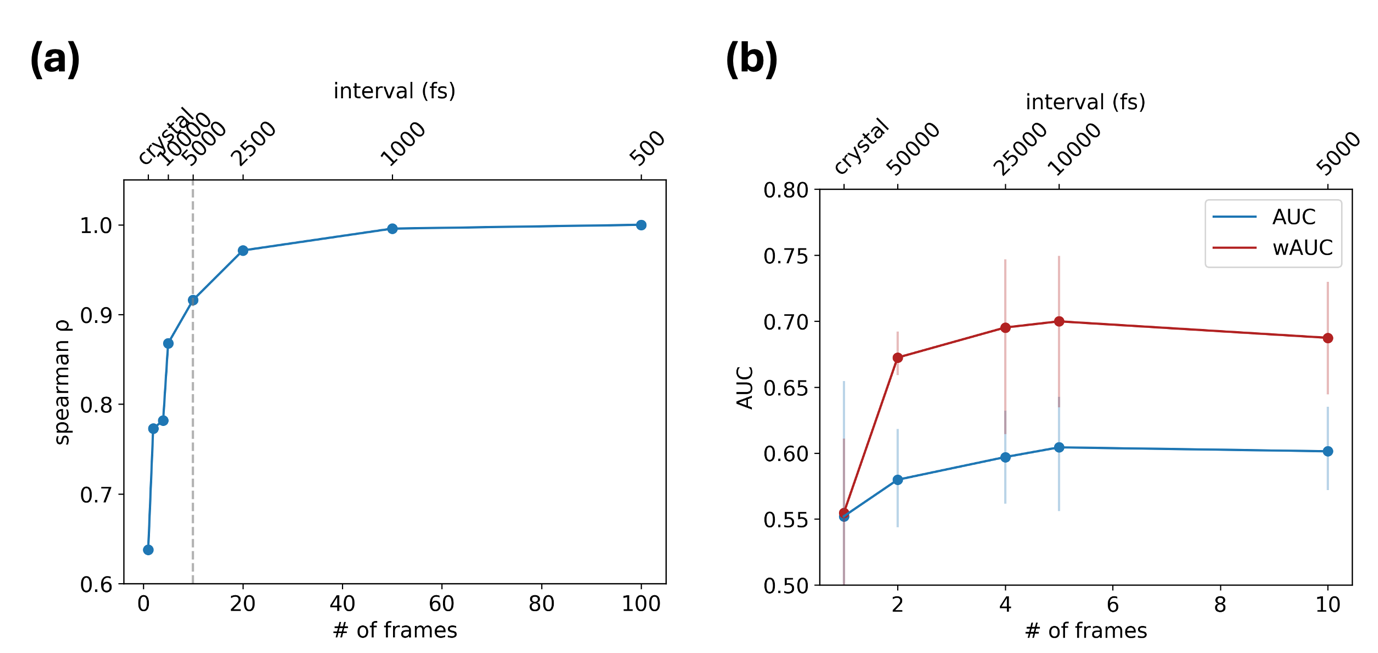


Figure S7 (a) Spearman ρ between variable output frequencies of the number of frames saved to a trajectory and an output frequency of 0.5 ps for all simulated mutations of ADH (n=397). The grey dotted line indicates the default FRESCO sampling rate (5000 fs/frame). (b) the effect of a lower sampling rate on the average weighted and unweighted ROC AUC for all experimentally tested mutations across all three proteins (n=216)). Especially the weighted AUC is affected at lower sampling rates, indicating the ranking of highly stabilizing mutations benefits from more dynamic data.

## Figure S8


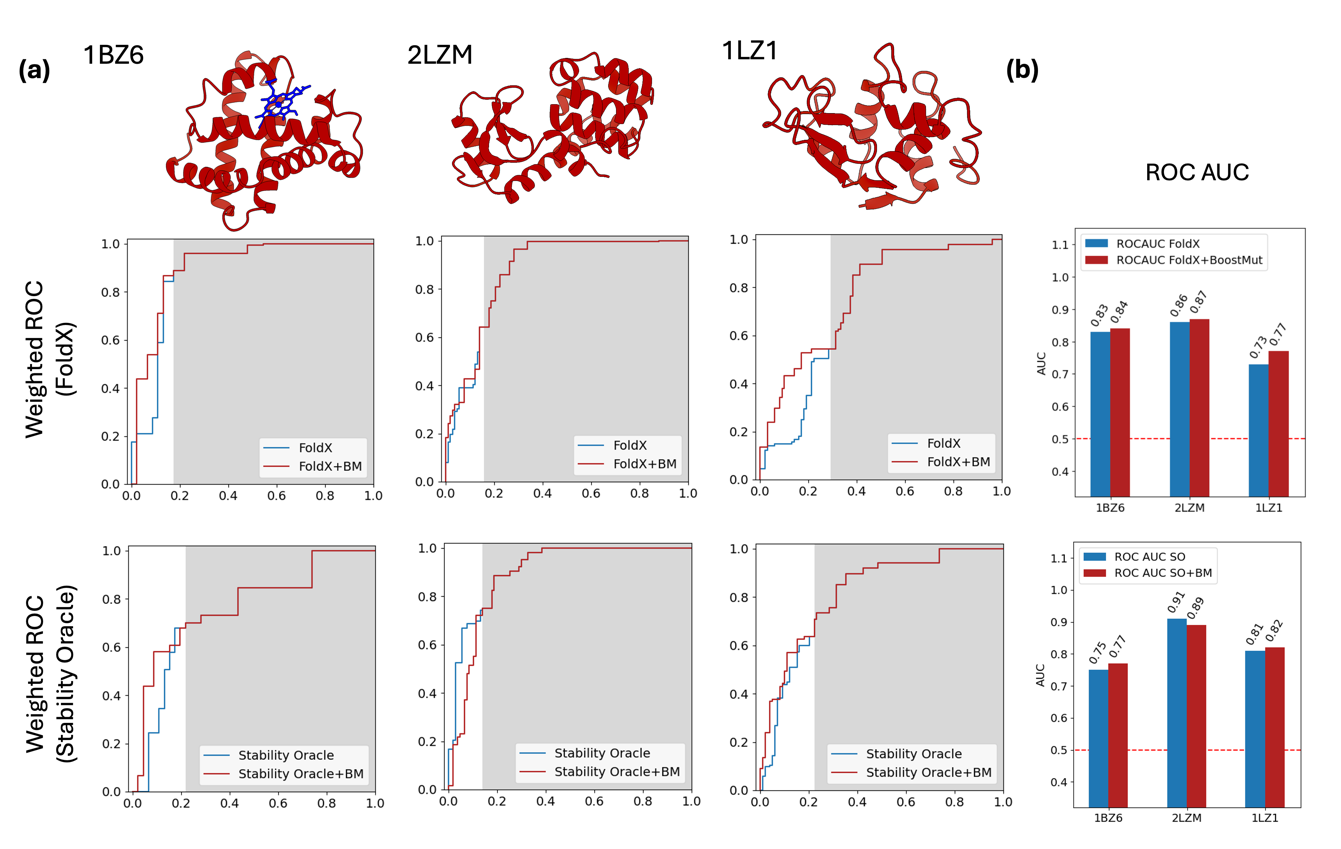


Figure S8 (a) Weighted ROC curves for three proteins from the T2837 test set, using either FoldX (top row) or Stability Oracle (bottom row) as the primary predictor. All mutations with a predicted ΔΔG < 0 were scored with the BoostMut (the area shown in white), all mutations with a predicted ΔΔG > 0 were scored by the primary predictor (the area shown in grey). (b) For all cases apart from Stability Oracle when used on 2LZM, the addition BoostMut resulted in a small increase in the weighted ROC AUC.
